# *Ent*-Clerodane Diterpenes from the Bark of *Croton oligandrus* Pierre ex Hutch. and Assessment of Their Cytotoxicity against Human Cancer Cell Lines

**DOI:** 10.3390/molecules23020410

**Published:** 2018-02-13

**Authors:** Stephanie Tamdem Guetchueng, Lutfun Nahar, Kenneth James Ritchie, Fyaz Mahmood Daud Ismail, Andrew Robert Evans, Satyajit Dey Sarker

**Affiliations:** Medicinal Chemistry and Natural Products Research Group, School of Pharmacy and Biomolecular Sciences, Faculty of Science, Liverpool John Moores University, Liverpool L3 3AF, UK; stevnyg@yahoo.fr (S.T.G.); L.Nahar@ljmu.ac.uk (L.N.); K.J.Ritchie@ljmu.ac.uk (K.J.R.); F.M.Ismail@ljmu.ac.uk (F.M.D.I.); A.R.Evans@ljmu.ac.uk (A.R.E.)

**Keywords:** *Croton oligandrus*, Euphorbiaceae, *ent*-clerodanes, crotonolin A, crotonolin B, cytotoxicity

## Abstract

New clerodane diterpenes, 12-*epi*-megalocarpodolide D (**2**) and an epimeric mixture of crotonolins A (**3**) and B (**4**), were isolated from the bark of *Croton oligandrus* following a bioassay-guided isolation protocol. Known compounds, megalocarpodolide D (**1**), 12-*epi*-crotocorylifuran (**5**), cluytyl-ferulate (**6**), hexacosanoyl- ferulate (**7**), vanillin (**8**), acetyl-aleuritolic acid (**9**) and lupeol (**10**), were also isolated. The structures of the isolated compounds (**1**–**10**) were elucidated by spectroscopic means. The cytotoxicity of compounds **1**–**10** was assessed against A549, MCF7, PC3 and PNT2 cell lines using the MTT assay. Compounds **1** and **2** showed moderate levels of activity against both A549 and MCF7 cells with **1** being the most active with IC_50_ values of 63.8 ± 13.8 and 136.2 ± 22.7 µM against A549 and MCF7 cells, respectively. The epimeric mixture of **3** and **4** was moderately active against A549 and PC3 cells (IC_50_ = 128.6 ± 31.0 and 111.2 ± 2.9 µM, respectively).

## 1. Introduction

**Figure 1:**
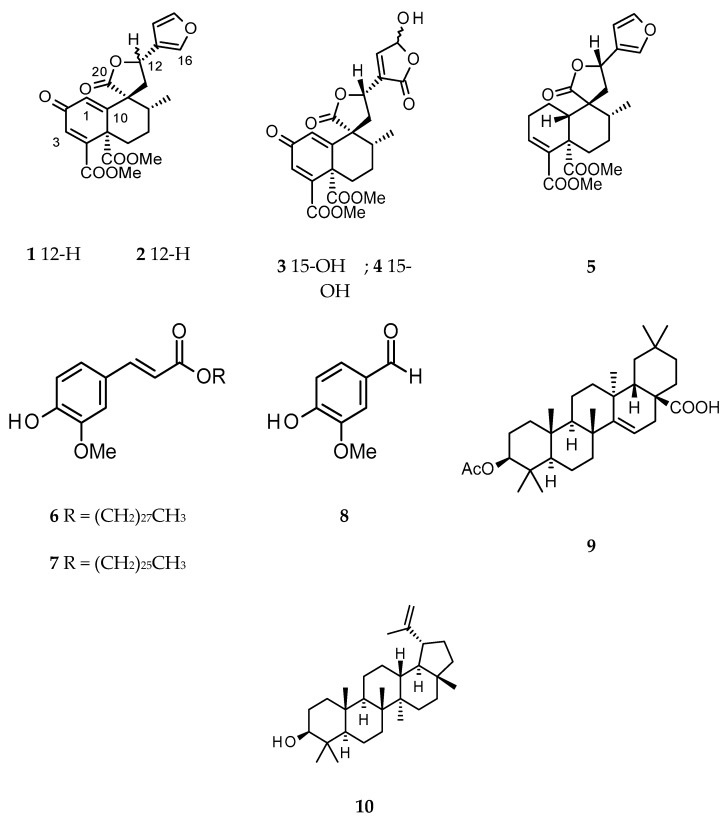
Chemical structures of compounds **1**–**10**.

The genus *Croton* L. belongs to the spurge family Euphorbiaceae, which is one of the largest families of flowering plants with about 300 genera and more than 7500 species [1]. This family has five subfamilies: Acalyphoideae, Crotonoideae, Euphorbioideae, Phyllanthoideae and Oldfieldoiideae [2]. The genus *Croton* L. belongs to the Crotonoideae subfamily, and comprises around 1300 species of herbs, shrubs and trees growing in tropical and subtropical regions of the globe [3]. The species of this genus have long been used in ethnomedicine. In the Ayurvedic system of medicine, *Croton oblongifolius* Roxb. and *Croton tiglium* L. have been mentioned for their use in the treatment of liver diseases, sprains and snake bites, as well as a purgative, since 2000 BC [4]. *Croton tiglium* has also been used in Chinese Traditional Medicine (TCM) to treat severe constipation. Popular traditional uses of *Croton* species include their use in the treatment of cancer, constipation, diabetes, fever, hypercholesterolemia, hypertension, inflammation, malaria, pain and weight-loss [5,6,7,8,9,10]. *Croton oligandrus* Pierre ex Hutch. is a tree (5–10 m high), commonly found in Western and Central African forests, especially in Cameroon and Gabon [11,12]. The stem bark decoction of *C. oligandrus* is taken orally in Cameroon to treat anaemia, pneumonia and splenomegaly [12,13,14]. Agnaniet et al. identified linalool as the main constituent of the essential oil of the species collected in Gabon, and showed that the oil possessed neither good antioxidants nor antiradical activity [15]. Further, studies of the species harvested in Cameroon revealed the presence of clerodane-type diterpenes as the main class of metabolites [16]. Clerodane diterpenes are widely distributed within the genus *Croton*, and known to possess antifeedant, cytotoxic and antiprotozoal properties [17]. As part of our ongoing research into bioactive compounds from Cameroonian medicinal plants [18,19], we shall report on the isolation of three new clerodane diterpenes (**2**–**4**), together with known compounds **1** and **5**–**10** (Figure 1), and assess their cytotoxicity against human cancer cell lines.

## 2. Results and Discussion

The *n*-hexane, DCM and MeOH extracts obtained from the bark of *C. oligandrus* were screened for their cytotoxic activity (Table 1). While the MeOH extract was devoid of any cytotoxicity below 250 µg/mL, the *n*-hexane extract displayed cytotoxicity against the cell lines, PC3 (human prostate cancer) and MCF7 (human breast adenocarcinoma), with IC_50_ values of 71.7 ± 1.5 and 31.5 ± 0.9 µg/mL, respectively, and the DCM extract was only active against the MCF7 cell line (IC_50_ = 59.7 ± 3.0 µg/mL). Fractionation of the active crude extracts followed by their cytotoxic evaluation identified fractions H1, H2, H4 and H5 of the *n*-hexane extract and fractions D1, D2, D3, D4 and D6 from the DCM extract as cytotoxic (Table 1).

Phytochemical analysis of the active fractions afforded a mixture (1:1) of ferulate derivatives, cluytyl-ferulate and hexacosanoyl-ferulate (**6** and **7**) [20,21], vanillin (**8**) [22], acetyl-aleuritolic acid (**9**) and lupeol (**10**) [23,24] from the fractions H2, H4 and H5. From the fractions D2 and D3, the mixture of **6** and **7** was also obtained by recrystallization and the remaining filtrate was found to be a mixture of **9** and **10**. Fractions D4 and D6 were purified by preparative HPLC to afford the known clerodane diterpenes megalocarpoidolide D (**1**) and 12-*epi*-crotocorylifuran (**5**) [25], and the new clerodanes 12-*epi*-megalocarpoidolide D (**2**), and crotonolins A (**3**) and B (**4**) (Figure 1). Fractions D1 and H1 showed a single spot on TLC, but this was found to be a mixture of fatty acids by preliminary ^1^H NMR and was not further purified.

Compounds **1** and **2** were isolated as white amorphous powders. Their molecular formula C_22_H_22_O_8_ was determined from the *pseudo* molecular ion peak at *m*/*z* 432.1650 calculated 432.1653 for C_22_H_22_O_8_NH_4_ [M + NH_4_]^+^, from the HRMS spectrum obtained in positive ion mode. The IR spectrum of **1** displayed absorption stretching bands at 1715, 1767 and 1663 cm^−1^ corresponding to the carbonyl of ester, lactone and ketone groups, respectively. Assignment of ^1^H and ^13^C NMR data of **1** (Table 2) was confirmed by its 2D NMR (COSY, HSQC and HMBC) data, and all data were in agreement with that of the *β*-substituted furanoclerodane, megalocarpoidolide D (**1**), previously isolated from the roots of *C. megalacarpoides* (*C. megalocarpus* Hutch.) [25]. In addition, in the NOESY experiment, a strong correlation was observed between H-12 and H-1 which confirmed the C-12 relative absolute configuration in **1** to be 12*S* as published for megalocarpoidolide D (**1**) [25]. Thus, compound **1** was identified as the known compound megalocarpoidolide D (**1**) (Figure 1).

The ^1^H and ^13^C NMR data of compound **2** (Table 2) were similar to those of **1**, but with slight differences (see Supplementary Material). Interpretation of ^1^H, ^13^C, COSY, HSQC and HMBC NMR spectral data of **2** concluded that its core structure was identical to that of **1**. However, the NOESY spectrum showed a strong correlation between H-12 and H-17 (instead of H-1 as in compound **1**), establishing the relative configuration at C-12 in **2**—the opposite to that of compound **1**. The absolute configuration at C-12 in **2** could be confirmed as 12*R* instead of 12*S* (as in **1**). Thus, compound **2** was identified as a new C-12*R* epimer of **1** and named 12-*epi*-megalocarpoidolide D (**2**) (Figure 1).

Compound **3** and its 15-epimer **4** were isolated as a white amorphous powder mixture with a molecular formula of C_22_H_22_O_10_, as determined from the HRMS data obtained in the negative ion mode (*m*/*z* 445.1142 [M − H]^−^ calculated 445.1140 for C_22_H_21_O_10_). The IR absorption bands at 3465, 1775, 1712 and 1661 cm^−1^ could be assigned to the stretch signals of hydroxyl and the carbonyls of ester, lactone and ketone groups, respectively. The assignment of the ^1^H and ^13^C NMR data (Table 2) was confirmed by the COSY, HSQC and HMBC spectral data analyses. A total of 22 carbon signals were observed in the ^13^C NMR spectrum (Table 2). The signals could be assigned to one methyl C-17 (δ 17.1), two methoxyls at δ53.3 and 53.7, six methines including three olefinic carbons at δ129.0, 131.7 and 149.8, ten quaternary carbon signals including an unsaturated ketone at δ187.2 (C-2) and the carbonyls of two esters at δ166.7 and 168.4 corresponding to C-18 and C-19, respectively. In the ^1^H NMR spectrum (Table 2), the 1H doublet at δ5.72 (*J* = 11.1, 5.5 Hz) could be assigned to H-12 and the two 1H multiplets at δ2.80 and 2.93, coupling with each other and with H-12 as evident from the COSY experiment, were assigned to the C-11*ax* and C-11*eq* protons, respectively. The doublet at δ1.15 (3H, *J* = 5.8 Hz) was assigned to C-17 and the corresponding coupled methine signal at δ1.82 was assigned to C-8. The C-6 and C-7 methylene protons were assigned to protons resonating at δ1.51 (ddd, *J* = 4.0, 13.5, 17.5 Hz), 3.00 (m) and 1.66 (m), 2.66 ppm (m), respectively. The olefinic protons at δ6.91 (d, *J* = 1.2 Hz) and 6.80 (d, *J* = 1.2 Hz) were assigned to protons C-1 and C-3, respectively. This assignment was consistent with the different correlations observed in the HMBC experiment. The ^1^H and ^13^C NMR data of **3** and **4** were similar to those of compound **1** and **2**, but there were no signals for protons of the furan ring, suggesting that the furan rings in **3** and **4** were modified. In the ^1^H NMR spectrum (Table 2), there were two doublets at δ6.19 and 7.40 for methines showing cross peak correlation in the HSQC spectrum with carbon signals at δ 99.3 and 149.8, respectively. These two methines, in the HMBC experiment, showed a strong correlation with a deshielded carbon signal at δ170.9. The above NMR data were similar to those of the modified furan ring present in salvidinin B, a clerodane diterpene isolated from *Salvia divinorum* [26]. The hemiacetal carbon C-15 was then attributed to the signal at δ99.3 ppm, the olefinic carbon C-14 at δ149.8 ppm, the carbonyl C-16 at δ170.9 ppm and C-13 at δ135.0 ppm. These attributions were supported by the correlation between the proton signal at δ7.40 (H-14) and the carbon C-12, and correlations between protons H-12 and H-11eq with C-13 observed in the HMBC experiment. However, the signals attributed to protons H-14 and H-15 were not broad singlets like in similar molecular cases [26,27,28]. In addition, the two protons did not show any correlation in the COSY experiment and the coupling constants of the two doublets were different (6.5 and 9.4 Hz). Therefore, it was clear that two broad singlets instead of doublets were present; this led to the conclusion that the obtained powder was a mixture of 15*α*-OH and 15*β*-OH isomers. Furthermore, analysis of the NOESY spectrum showed a strong correlation between H-12 and H-1, suggesting a C-12*S* configuration (Figure 1). In addition, the optical inactivity of the mixture indicated that it was a racemic mixture. Thus, the compounds were then identified as 2-oxo-15α-hydroxy-18,19-dimethoxycarbonyl-15,16-epoxy-*ent*-cleroda-1(10),3,13(16),14-tetraen-20,12-olide and 2-oxo-15β-hydroxy-18,19-dimethoxycarbonyl-15,16-epoxy-*ent*-cleroda-1(10),3,13(16),14-tetraen-20,12-olide and two clerodane diterpenes not previously described, which were given the trivial names crotonolins A (**3**) and B (**4**), respectively. 

In addition to the genus *Croton*, clerodanes, a large group of bicyclic 20-carbon terpene compounds, are also found in the genera *Ajuga*, *Gymnocolea*, *Jateorhiza*, *Scutelaria*, *Teucrium*, *Tinospora* and *Zuelania* [17]. The distribution of clerodane diterpenes and their chemotaxonomic implications, as well as biological activities, have been well-documented by Li et al. [17]. 

Compounds **1**–**10** were evaluated for their cytotoxicity against A549 (adenocarcinomic human alveolar basal epithelial), MCF7 (human breast adenocarcinoma), PC3 (human prostate cancer) and PNT2 (human normal prostate epithelium) cells. The concentrations which inhibited fifty percent of cell growth after treatment with each compound are presented in Table 1. Doxorubicin was used as a positive control, with IC_50_ ranging from 0.7 to 16.4 µM between the cell lines used. Compounds **3**–**10** were inactive against the MCF7 cell line. Compounds **1** and **2** exhibited moderate levels of cytotoxicity against both A549 and MCF7 cells with **1** being the most active with IC_50_ values of 63.8 ± 13.8 and 136.2 ± 22.7 µM against A549 and MCF7 cells, respectively. The epimeric mixture **3** and **4** was moderately active against A549 and PC3 cells (IC_50_ = 128.6 ± 31.0 and 111.2 ± 2.9 µM, respectively). All these compounds displayed reduced levels of cytotoxicity compared with those of the corresponding crude extracts and fractions. Consistent with this finding, it has been demonstrated previously that the cytotoxicity of an extract may be a consequence of the synergetic action of all the compounds present in that extract and not due to the action of a single compound [29]. Moreover, in a number of previous studies, several plant-derived clerodane diterpenes showed good cytotoxic activity against various cancer cell lines [30,31,32,33,34].

## 3. Material and Methods

### 3.1. General

Chromatographic solvents were purchased from Fisher Scientific, Loughborough, UK, and used without further purification. Silica gel (70–230 mesh) and silica gel 60H, purchased from Sigma-Aldrich, Gillingham, Dorset, UK, were used for open column chromatography (CC) and vacuum liquid chromatography (VLC), respectively. Analytical TLC was carried out on 0.2 mm Sigel 60 F_254_ plates (Merck, Darmstadt, Germany). Spots were visualized under short (254 nm) and long wavelengths (366 nm), as well as by spraying them with a 1% anisaldehyde solution in aqueous H_2_SO_4_, followed by heating to 105 °C for 5 min. The NMR spectroscopic analyses were performed on a Bruker AMX600 NMR spectrometer, Coventry, UK (600 MHz for ^1^H, and 150 MHz for ^13^C). MS analyses were performed on Xevo G2-S ASAP or LTQ Orbitrap XL 1 spectrometers, Swansea, UK. HPLC-DAD analysis was performed on an Agilent 1260 Infinity series, Stockport, UK. Extracts and fractions were analyzed on a Phenomenex Gemini-NX 5 U C_18_ column (150 × 4.6 mm, 5 μm, Phenomenex, Macclesfield, NC, USA). An ACE preparative column (150 × 21.2 mm, 5 μm, Hichrom Ltd., Reading, UK) was used for isolating compounds. A gradient 30–100% MeOH (0.1% TFA) was used in water over 30 min, with a flow rate of 1 mL/min and 10 mL/min for analytical and preparative HPLC, respectively. The column temperature was set at 25 °C. The chromatogram was used to monitor variable UV–Vis wavelengths (215, 254, 280 and 320 nm). Optical rotation was determined using a Bellingham–Stanley ADP660 polarimeter, Kent, UK (MeOH, c in g/100 mL). UV spectra were recorded on an Analytik Jena Specord 210 spectrophotometer, Jena, Germany. IR spectra were recorded on an Agilent Cary 630 FT-IR, Stockport, UK.

### 3.2. Plant Material 

The bark of *Croton oligandrus* Pierre ex Hutch. was collected from the Mount Eloundem, Centre Region, Cameroon, in June 2015, and identified by Mr. Victor Nana, a retired taxonomist at the Cameroon National Herbarium, where a voucher specimen (6687/SFR) was deposited. 

### 3.3. Extraction and Isolation 

The air-dried and ground bark (330.0 g) of *C. oligandrus* were extracted, successively, with *n*-hexane, DCM and MeOH using a Soxhlet extractor (800 mL, 10 cycles each). After evaporation at 40 °C under reduced pressure, 3.7 g, 2.6 g and 7.4 g of *n*-hexane, DCM and MeOH extracts were obtained, respectively. The crude extracts were screened against three cancer cell lines: breast (MCF7), prostate (PC3) and lung (A549) cancer cells. DCM and *n*-hexane extracts were found to be the most active and were submitted to further fractionation using VLC. A portion of the *n*-hexane (3.3 g) and DCM (2.2 g) extracts were adsorbed onto normal silica gel (70–230 mesh) and loaded on the top of a VLC column pre-packed with silica gel 60H. A vacuum was applied and the column was eluted with a stepwise gradient of mobile phase, consisting of an increasing amount of ethyl acetate (EA) in *n*-hexane (Hex/EA 0%, 10%, 20%, 40%, 60% and 80%) or MeOH in DCM (DCM/MeOH 0%, 2%, 6%, 10%, 15% and 25%) for the *n*-hexane and DCM extracts, respectively, to obtain six different fractions. The fractions were also screened against the cell lines cited above. Fractions H1, H2 and H4 of *n*-hexane extract and D2, D3, D4, and D6 of DCM extracts showed potent cytotoxic activities and were subjected to further purification. H2 (420.9 mg) of *n*-hexane extract was purified using a CC over silica gel to give an equimolar mixture (18.2 mg) of **6** and **7**, and acetyl aleuritolic acid **9** (4.4 mg). Lupeol **10** (6.1 mg) and vanillin **8** (9.0 mg) were obtained from D4 by recrystallization. Ferulate derivatives, a mixture of **6** and **7** (3.2 mg), were obtained from F2 and F3 of the DCM crude extract by recrystallization. Preliminary ^1^H NMR of the remaining filtrate showed that this latter was a mixture of lupeol and acetyl aleuritolic acid, which was not purified further. D4 and D6 showed similar TLC and HPLC profiles and were mixed together for purification. The mixture of the two fractions (783.0 mg) was purified by preparative HPLC using an ACE Gemini-NX 5 U C18 column, Reading, UK (150 × 21.2 mm, Hichrom Ltd., UK), flow rate 10 mL/min, mobile phase gradient of water (A) and methanol (B) both containing 0.1% TFA: 30–100% B, 0–30 min; 100% B, 30–35 min; 100–30% B, 35–40 min, monitored at wavelengths of 254 and 280 nm to yield crotolins A (**3**) and B (**4**) (3.2 mg); 12-*epi*-megalocarpoidolide D (**2**) (6.8 mg), megalocarpoidolide D (**1**) (9.2 mg), and crotocorylifuran (**5**) (8.2 mg) were purified with the retention time (*t*_R_) 15.7, 18.8, 20.5 and 24.6 min, respectively.

### 3.4. 12-epi-Megalocarpoidolide D (***2***)

White powder (6.8 mg); HRMS *m*/*z* 432.1650 [M + NH_4_]^+^ (calc. for C_22_H_22_O_8_NH_4_, 432.1653) in positive ion mode; ${\left[\alpha \right]}_{D}^{25}$ + 81.7 (c 0.0018, MeOH); FT-IR (ATR) ν_max_ 1715, 1767 and 1663 cm^−1^. UV λ_max_ (MeOH) nm: 216, 224, 230, 252; see Table 2 for NMR data. 

### 3.5. Crotonolins A and B (***3*** and ***4***)

White powder (3.2 mg); HRMS *m*/*z* 445.1142 [M − H]^−^ (calc. for C_22_H_21_O_10_, 445.1140) in negative ion mode; ${\left[\alpha \right]}_{D}^{25}$ 0.0 (c 0.009, MeOH); FT-IR (ATR) ν_max_ 3465, 2932, 1775, 1712 and 1661 cm^−1^; UV λ_max_ (MeOH) nm; 218, 222, 230, 252; see Table 2 for NMR data.

### 3.6. Cell Viability Assay

The in vitro activity of the crude extracts, fractions and isolated compounds from *C. oligandrus* on metabolic cell viability was assessed against A549 (adenocarcinoma human alveolar basal epithelial cell 211 line), MCF7 (human breast adenocarcinoma cell line), PC3 (human prostate cancer cell line) and PNT2 212 (human normal prostate epithelium cell line). The resulting inhibitory activity is termed “cytotoxic activity” throughout the manuscript. The cell lines were grown in RPMI medium supplemented with l-glutamine (2 mM), penicillin (100 U/mL), streptomycin (100 μg/mL) and 10% foetal bovine serum (FBS) and cultured at 37 °C, 5% CO_2_ and 95% humidity. For experimental use, the cells were seeded into 96-well plates (1.2 × 10^4^/well) and incubated for 24 h. Cells were then treated with crude extract (0–250 µg/mL) for 24 h or isolated compounds (0 to 200 µM) for 48 h and the cell viability was measured using the MTT assay [35]. The formazan crystals formed were dissolved in DMSO and optical density was read at 570 nm using a ClarioStar plate reader, Manchester, UK. Three individual wells were assayed per treatment; the assay was repeated three times and cytotoxic activity was determined using the percentage of absorbance compared to the control cells [(absorbance of treated cells/absorbance of untreated cells) × 100]. Doxorubicin was used as the positive control and the IC_50_ value of each test sample was calculated using the software Graphad Prism 7.02, La Jolla, CA, USA.

## 4. Conclusions

In the present study, crude extracts, fractions and isolated compounds from *C. oligandrus* bark were evaluated for their cytotoxicity against a panel of cancer cells lines; A549, MCF7, PC3 and PNT2. Bioassay-guided isolation carried out on active fractions using a combination of open column chromatography, recrystallization and preparative HPLC resulted in the identification of new clerodane diterpenes, 12-*epi*-megalocarpoidolide D (**2**), crotonolins A (**3**) and B (**4**), together with the known compounds megalocarpoidolide D (**1**), 12-*epi*-crotocorylifuran (**5**), cluytyl-ferulate (**6**), hexacosanoyl-ferulate (**7**), vanillin (**8**), acetyl-aleuritolic acid (**9**) and lupeol (**10**). All tested compounds, including the new clerodanes **2**–**4**, were inactive or showed at best, a marginal level of cytotoxicity against A549, MCF-7 and PC-3 cancer cells, but were also nontoxic to PNT2 normal prostate epithelium cells.

## Figures and Tables

**Table 1:** Cell growth inhibitory activities of the extracts, fractions and isolated compounds against cancer and noncancerous (PNT2) cells *.

	IC_50_ Values			
**Extracts/Fractions (µg/mL)**	**A549**	**MCF-7**	**PC-3**	**PNT2**
Hexane extract	>250	71.7 ± 1.5	31.5 ± 0.9	nd
DCM extract	>250	59.7 ± 3.0	>250	nd
MeOH extract	>250	>250	>250	nd
D1	39.5 ± 2.9	106.0 ± 4.2	209.3 ± 7.6	nd
D2	54.9 ± 1.7	20.2 ± 1.2	39.9 ± 2.9	nd
D3	126.8 ± 1.2	52.5 ± 0.5	90.3 ± 0.6	nd
D4	44.4 ± 3.1	52.2 ± 0.3	104.3 ± 4.9	nd
D5	>250	>250	>250	nd
D6	>250	66.2 ± 1.5	>250	nd
H1	147.5 ± 1.5	84.5 ± 5.2	13.2 ± 0.0	nd
H2	102.2 ± 0.9	103.7 ± 4.9	109.3 ± 3.7	nd
H3	>250	173.1 ± 4.9	>250	nd
H4	106.6 ± 6.6	60.6 ± 1.2	211.8 ± 10.1	nd
H5	49.9 ± 5.5	42.7 ± 12.5	>250	nd
H6	>250	150.4 ± 5.9	>250	nd
**Compounds (µM)**				
**1**	63.8 ± 13.8	136.2 ± 22.7	>200	>200
**2**	138.6 ± 22.1	171.3 ± 51.4	>200	>200
**3/4**	128.6 ± 31.0	>200	111.2 ± 2.9	>200
**5**	106.6 ± 27.2	>200	>200	>200
**6/7**	>200	>200	160.9 ± 36.2	>200
**8**	>200	>200	>200	>200
**9**	136.8 ± 18.9	>200	172.3 ± 39.7	167.5 ± 25.3
**10**	>200	>200	135.6 ± 21.1	>200
Doxorubicin	1.3 ± 0.3	0.7 ± 0.1	16.4 ± 2.9	1.5 ± 0.3

* Data are represented as mean ± SEM (*n* = 3); IC_50_ = sample concentration that caused 50% cell growth inhibition; nd = not determined.

**Table 2:** ^1^H and ^13^C NMR data ^a^ of diterpenes **1**–**4**.

Position	Chemical Shift in ppm					
	^1^H (coupling constant *J* in Hz)			^13^C		
	1 ^b^	2 ^b^	3 + 4 ^c^	1 ^b^	2 ^b^	3 + 4 ^c^
1	6.47 br d (1.2), 1H	6.47 d (1.0), 1H	6.91 d (1.2), 1H	127.8	129.1	129.0
2	-	-	-	185.8	185.7	187.2
3	6.78 d (1.3), 1H	6.78 d (1.0), 1H	6.80 d (1.2), 1H	131.4	130.9	131.7
4	-	-	-	150.7	151.3	152.4
5	-	-	-	53.5	55.1	54.3
6	1.45 ddd (3.7, 13.3, 17.5), 1H	1.44 ddd (9.3, 13.5, 17.7), 1H	1.51 ddd (4.0, 13.5, 17.5), 1H	33.1	33.2	33.9
	3.11 dt (3.0, 6.1), 1H	3.12 dt (3.3, 13.3), 1H	3.00 m, 1H			
7	1.71 m, 1H	1.65 m, 1H	1.66 m, 1H	26.5	27.2	27.5
	2.78 m, 1H	2.49 ddd (4.2, 13.9, 17.1), 1H	2.66 m, 1H			
8	1.75 m, 1H	1.78 m, 1H	1.82 m, 1H	39.7	43.7	40.0
9	-	-	-	55.0	53.6	55.9
10	-	-	-	155.4	155.7	156.2
11	2.78 m, 1H	2.69 dd (8.2, 14.3), 1H	2.80 m, 1H	38.9	39.2	36.9
	2.65 dd (11.1, 14.5), 1H	2.94 dd (6.3, 14.2), 1H	2.93 m, 1H			
12	5.55 dd (5.2, 11.1), 1H	5.57 t (7.0), 1H	5.72 dd (5.5, 11.1), 1H	71.3	72.0	72.4
13	-	-	-	123.5	125.0	135.0
14	6.45 m, 1H	6.41 m, 1H	7.40 and 7.39 br s, 1H	108.1	107.8	149.8
15	7.47 br t (1.5, 3.1), 1H	7.49 br d (1.6), 1H	6.20 and 6.18 br s, 1H	144.4	144.6	99.3
16	7.54 br s, 1H	7.45 m, 1H	-	140.5	139.7	170.9
17	1.17 d (6.4), 3H	1.23 d (6.7), 3H	1.15 d (5.8), 3H	16.9	17.7	17.1
18	-	-	-	165.3	165.5	166.7
18-OMe	3.84 s, 3H	3.84 s, 3H	3.71 s, 3H	53.0	52.9	53.3
19	-	-	-	166.3	166.7	168.4
19-OMe	3.65 s, 3H	3.71 s, 3H	3.60 s, 3H	53.2	53.1	53.7
20	-	-	-	172.2	173.1	174.0

^a^ All assignments were confirmed unequivocally as based on COSY, HSQC, HMBC and NOESY experiments; Spectra obtained in: ^b^ CDCl_3_ (300 MHz for ^1^H and 75 MHz for ^13^C NMR, respectively) and ^c^ CD_3_OD (600 MHz for ^1^H and 150 MHz for ^13^C NMR, respectively).

## Supplementary Materials

The spectra of new compounds (**1**–**4**) are available online.
