# Supplementary material for: Ent-Clerodane Diterpenes from the Bark of Croton oligandrus Pierre ex Hutch. and Assessment of Their Cytotoxicity against Human Cancer Cell Lines
Source: Molecules. 2018 Feb 13;23(2):410. doi: 10.3390/molecules23020410 (PMC6017299; doi:10.3390/molecules23020410)
Supplement: Supplementary file 1 [file molecules-23-00410-s001.pdf]

COMPOUND 1

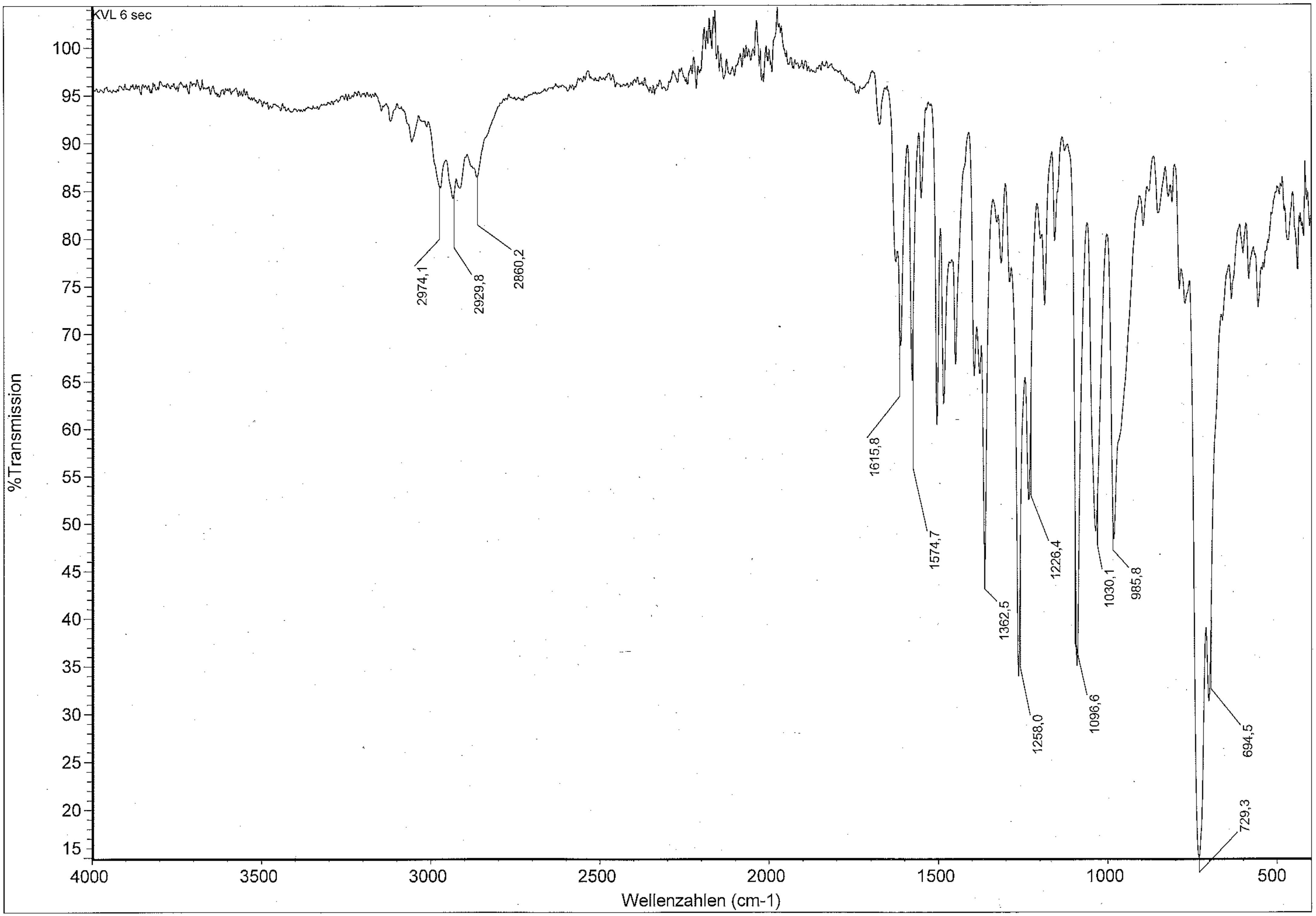
KVL 6 sec
%Transmission
Wellenzahlen (cm-1)
2974,1
2929,8
2860,2
1615,8
1574,7
1362,5
1258,0
1226,4
1096,6
1030,1
985,8
729,3
694,5

Analysis Report

Esquire 3000

Universität Bielefeld

Analysis Info

Analysis Name	OC3_KenmogneAr_0822_AKVL6_01.d
Method	Tune-nan.MS
Workgroup	OC3
Comment	Ar. Kenmogne, OC3, AKVL6, ACN

Operator	S.Heitkamp
Acquisition Date	24.08.2016 15:36:01
Print Date	24.08.2016 15:38:28

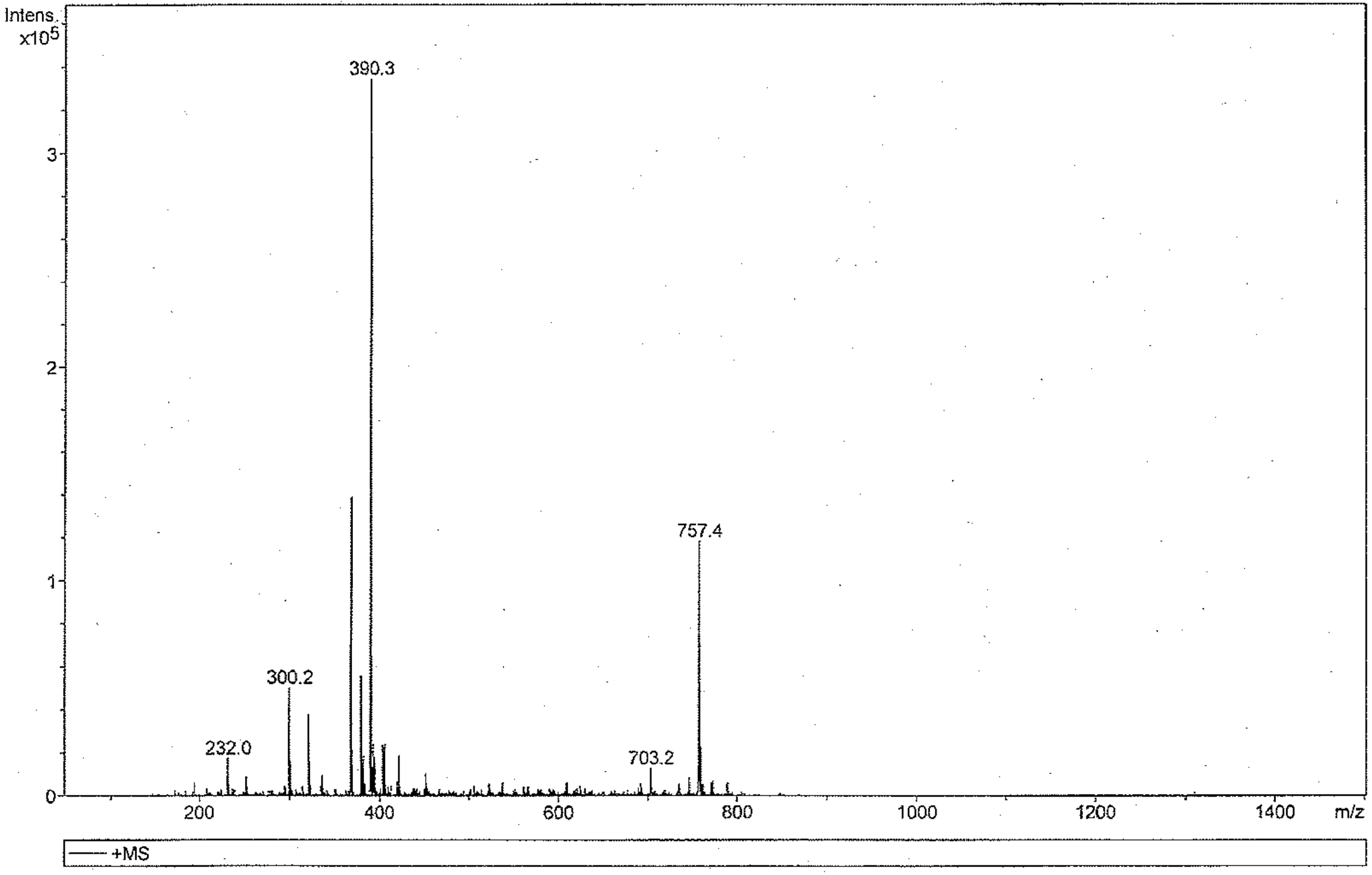

Acquisition Parameter

**Mode**	
Ion Source Type	NanoESI, off-line
Ion Polarity	Positive
Mass Range Mode	Std/Normal
Scan Begin	50 m/z
Scan End	1500 m/z
Averages	20 Spectra
Rolling	on
Rolling, Averages	2 cts
**Tune SPS**	
Target Mass	400 m/z
Compound Stability	40 %
Smart Parameter Setting	active
**Tune Instrument**	
Trap Drive	40.8
Octopole RF Amplitude	120.0 Vpp
Lens 2	-60.0 Volt
Capillary Exit	91.8 Volt
Skim 1	22.9 Volt
Skim 2	6.0 Volt
Lens 1	-5.0 Volt
Cap Exit Offset	68.9 Volt
Octopole	2.54 Volt
Octopole Delta	2.40 Volt
Dry Temp (Set)	100 °C
Nebulizer (Set)	2.00 psi
Dry Gas (Set)	2.00 l/min
Accumulation Time	666 µs
ICC Target	5000
Charge Control	on

OC3_KenmogneAr_0810_AKVL6.10.fid
Ar. Kenmogne, OC3, AKVL6
PROTON CDCl3 {C:\Bruker\TopSpin3.0} Service 53

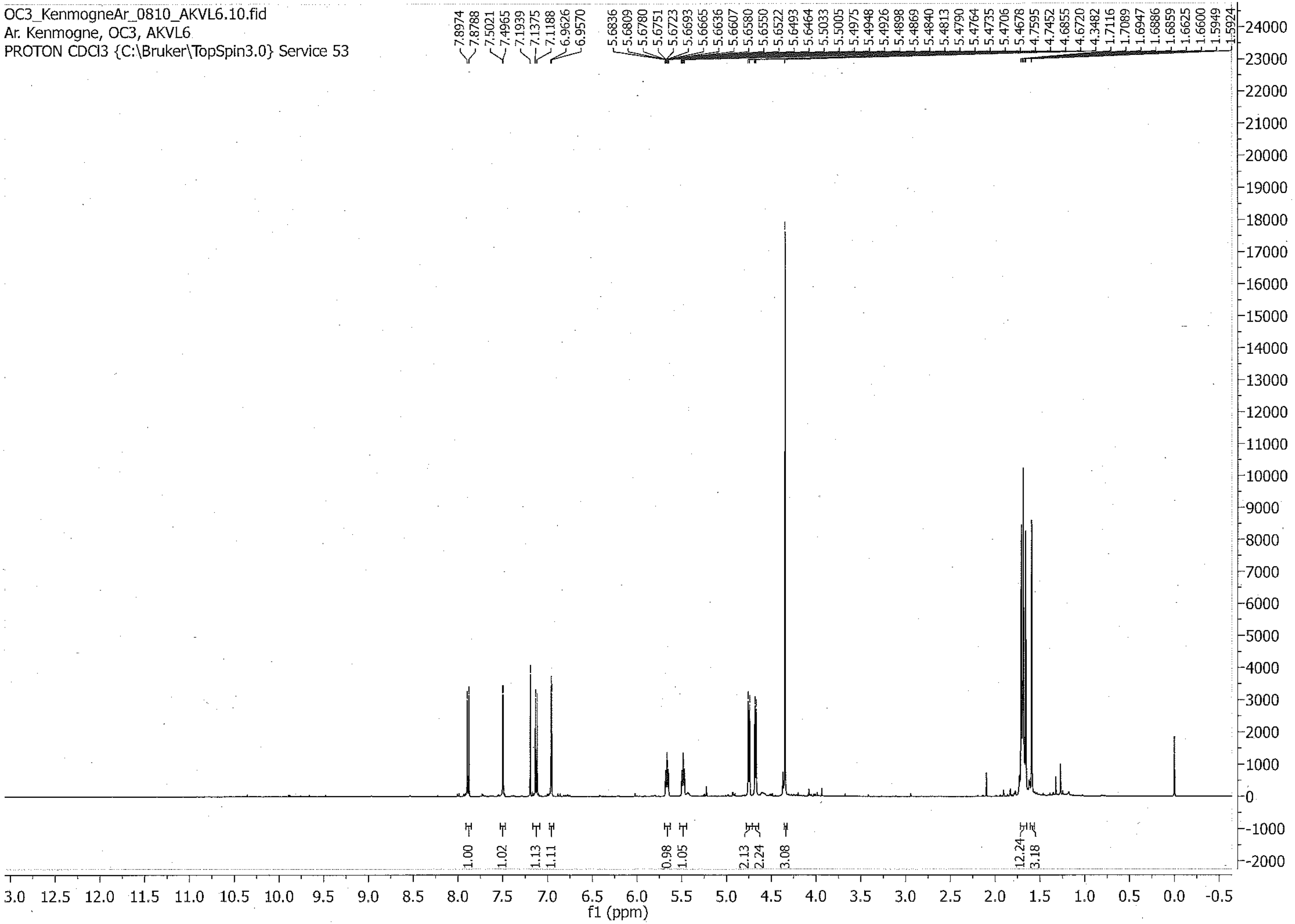

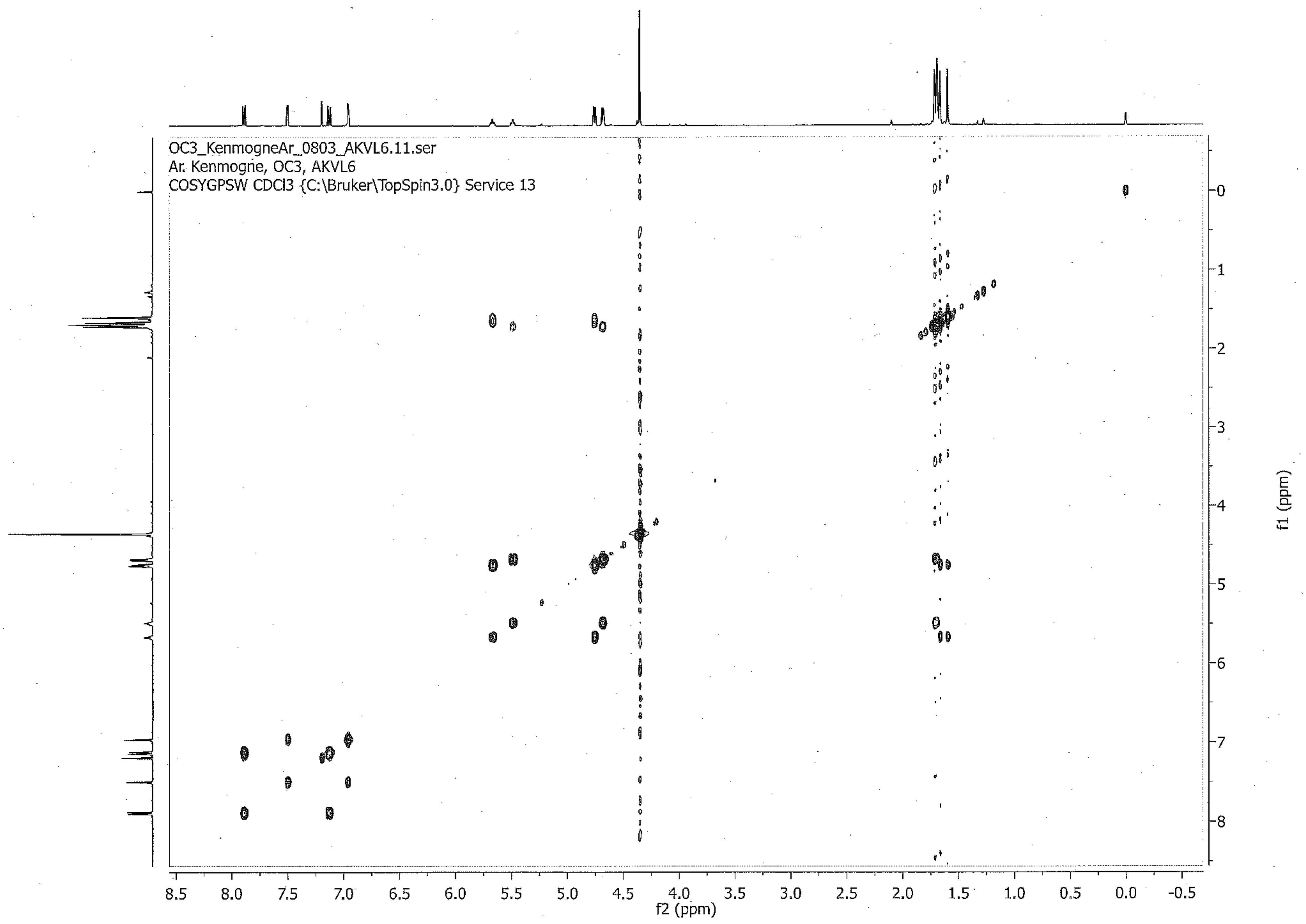
OC3_KenmogneAr_0803_AKVL6.11.ser
Ar. Kenmogne, OC3, AKVL6
COSYGPSW CDCl3 {C:\Bruker\TopSpin3.0} Service 13
f2 (ppm)
f1 (ppm)

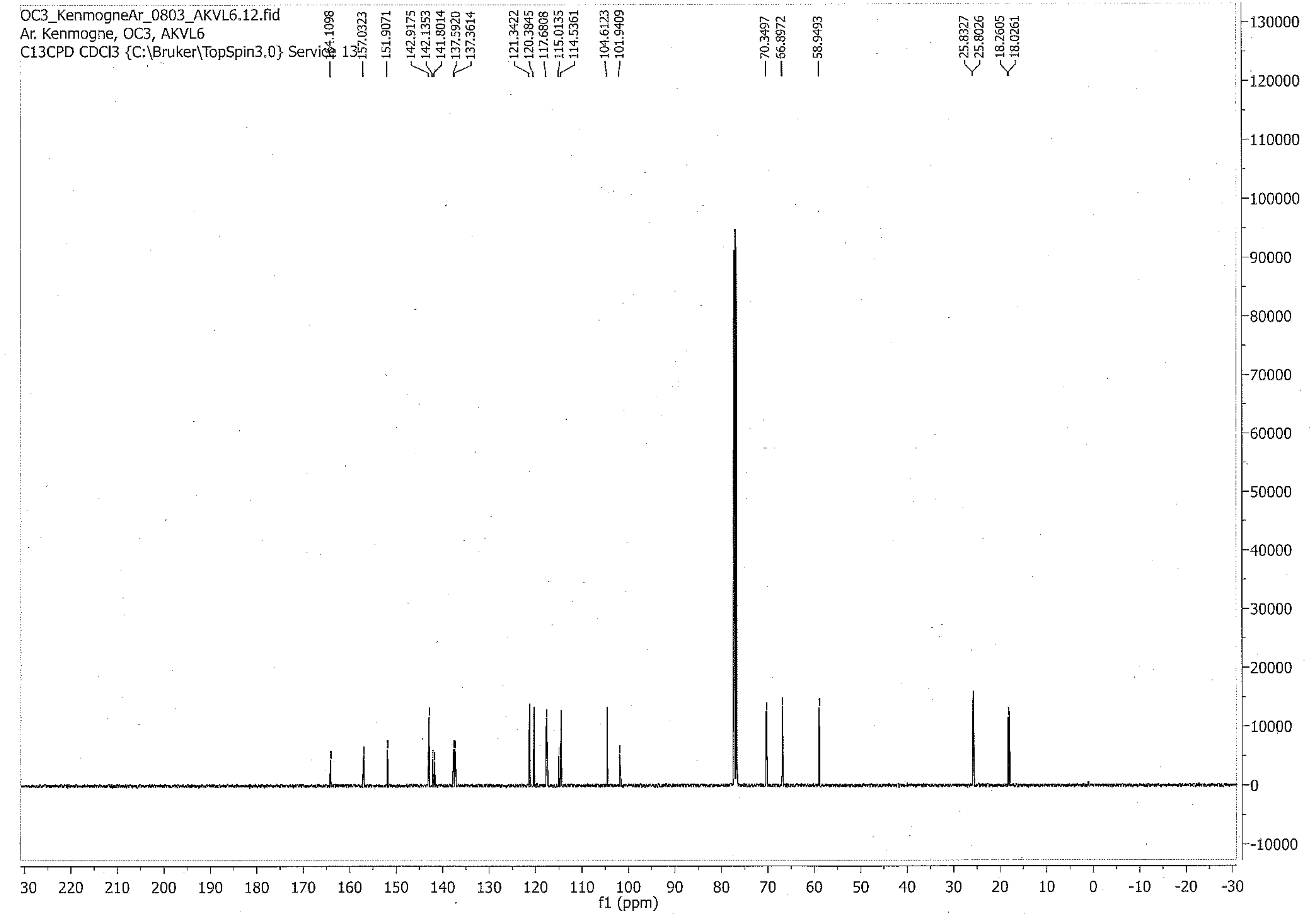

OC3_KenmogneAr_0803_AKVL6.12.fid
Ar. Kenmogne, OC3, AKVL6
C13CPD CDCl3 {C:\Bruker\TopSpin3.0} Service 13
164.1098
157.0323
151.9071
142.9175
142.1353
141.8014
137.5920
137.3614
121.3422
120.3845
117.6808
115.0135
114.5361
104.6123
101.9409
70.3497
66.8972
58.9493
25.8327
25.8026
18.2605
18.0261
f1 (ppm)

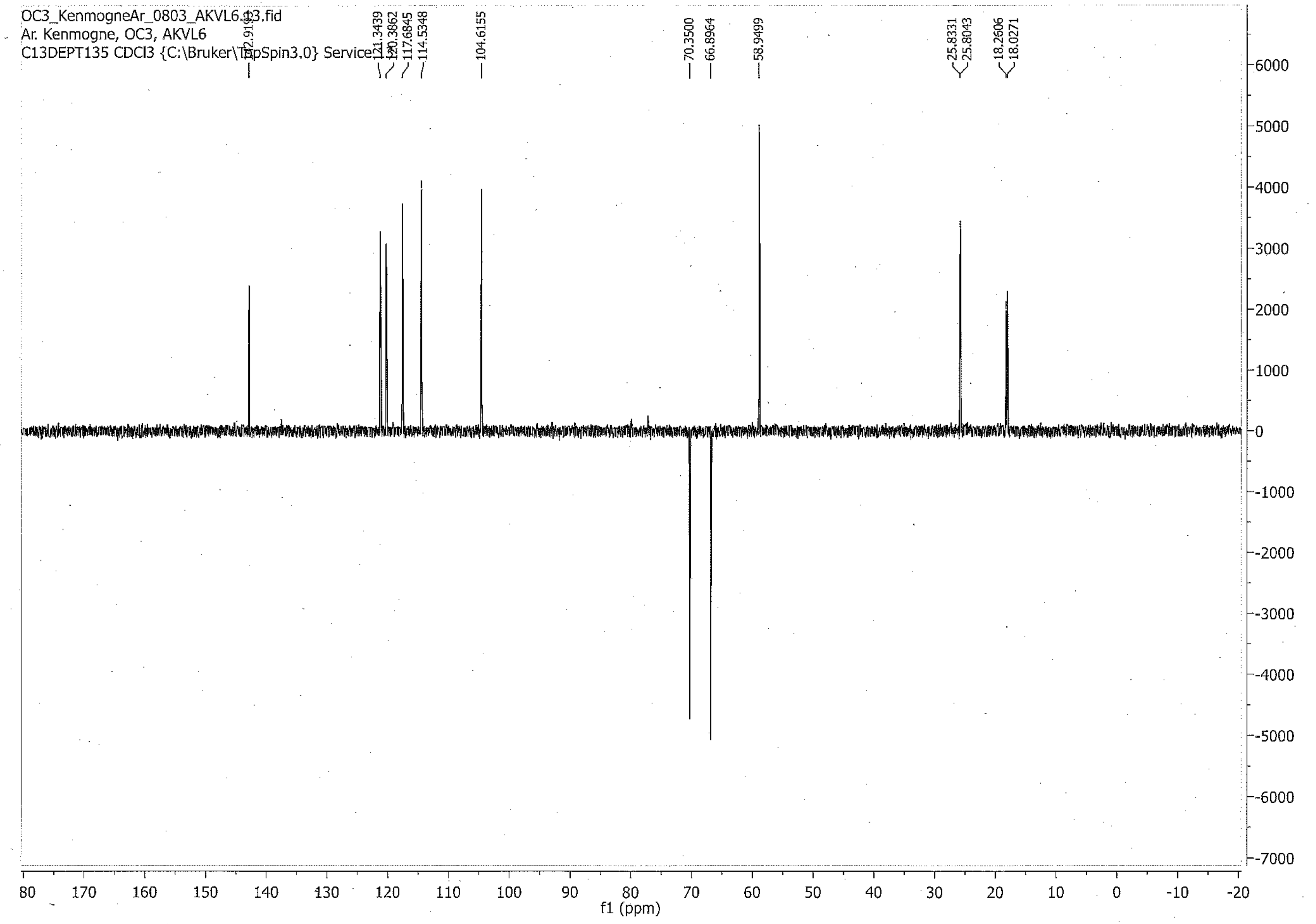
OC3_KenmogneAr_0803_AKVL6.13.fid
Ar. Kenmogne, OC3, AKVL6
C13DEPT135 CDCl3 {C:\Bruker\TopSpin3.0} Service
121.3439
120.3862
117.6845
114.5348
104.6155
70.3500
66.8964
58.9499
25.8331
25.8043
18.2606
18.0271
f1 (ppm)

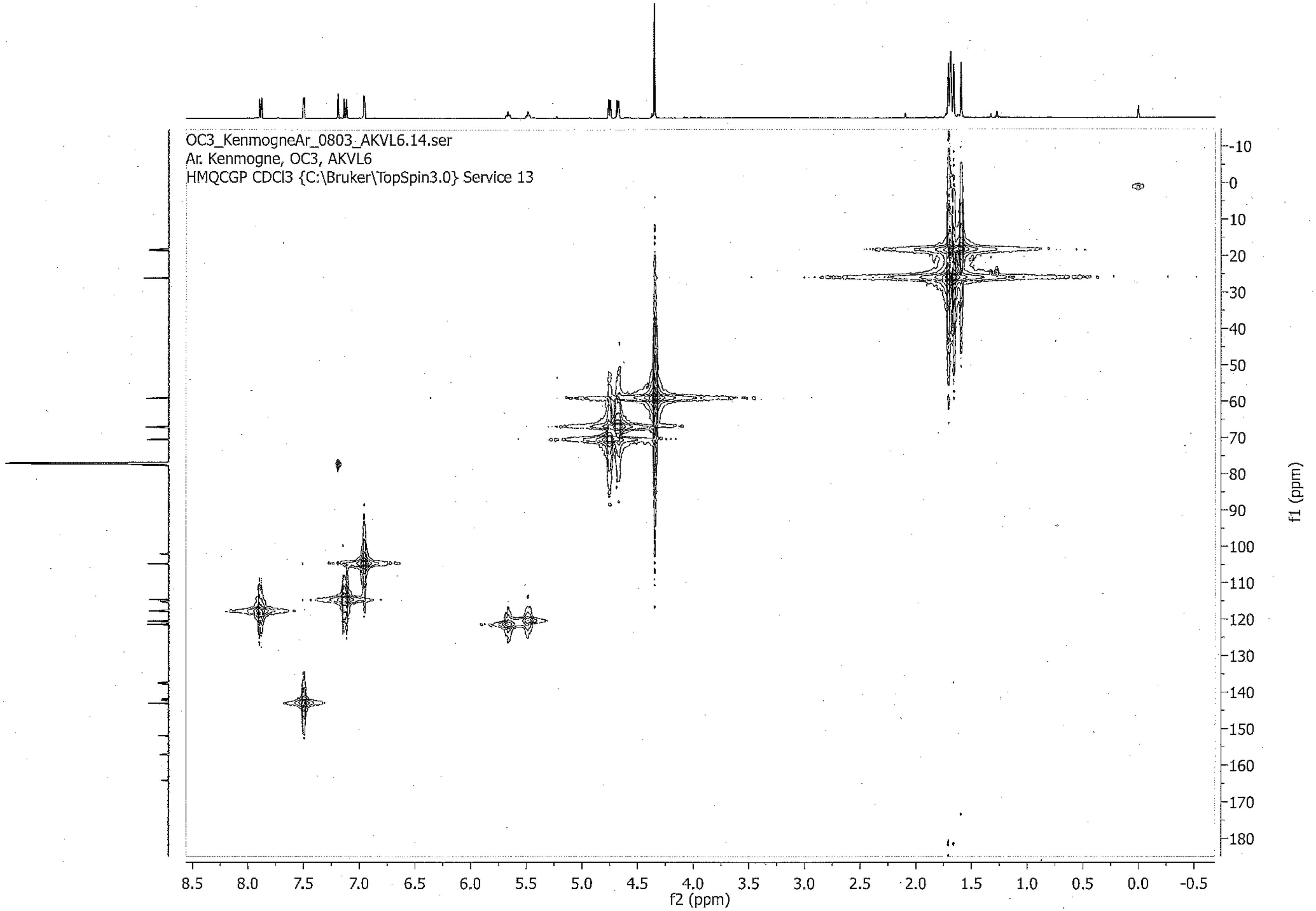
OC3_KenmogneAr_0803_AKVL6.14.ser
Ar. Kenmogne, OC3, AKVL6
HMQCGP CDCl3 {C:\Bruker\TopSpin3.0} Service 13
f2 (ppm)
f1 (ppm)

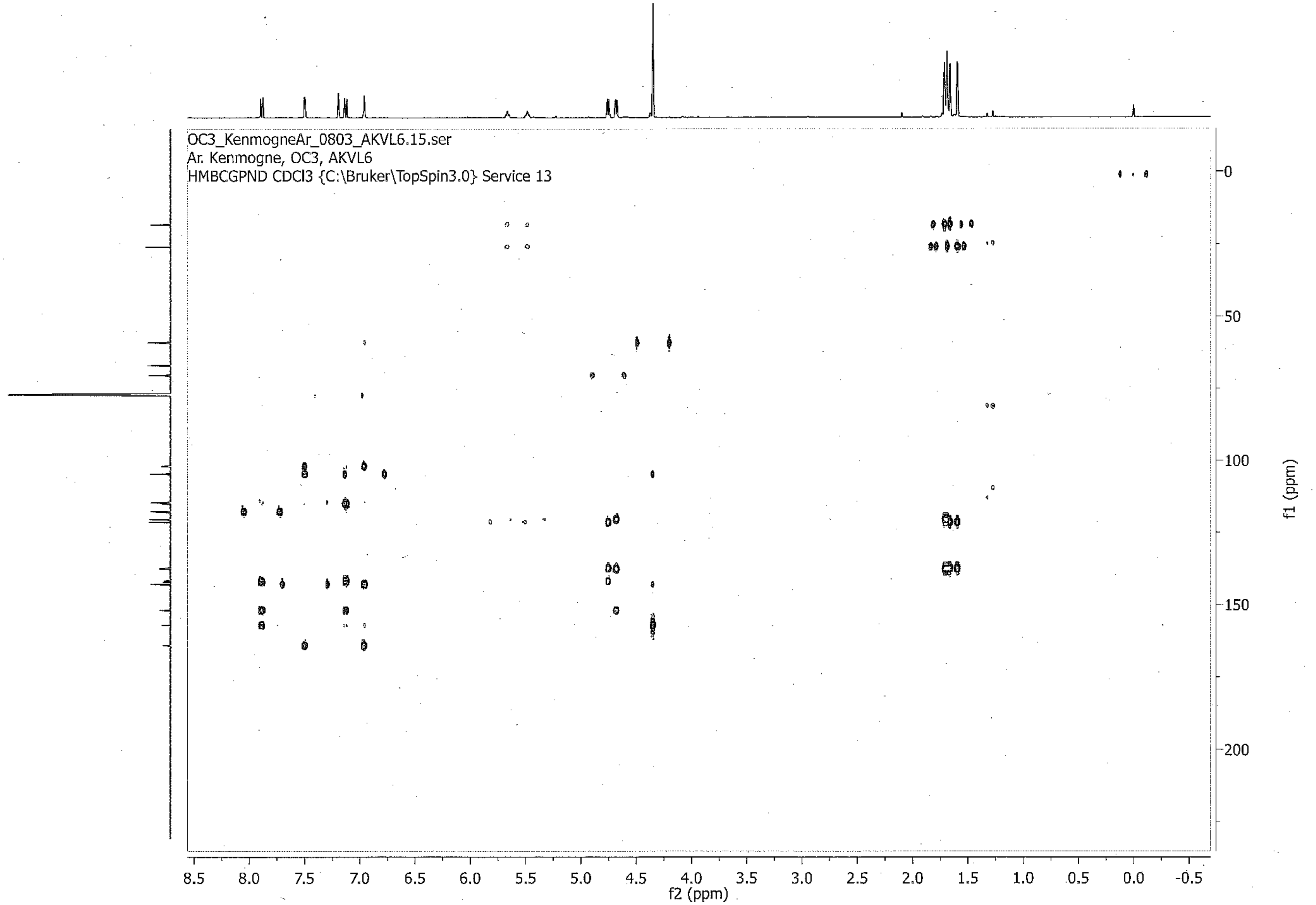
OC3_KenmogneAr_0803_AKVL6.15.ser
Ar. Kenmogne, OC3, AKVL6
HMBCGPND CDCl3 {C:\Bruker\TopSpin3.0} Service 13
f2 (ppm)
f1 (ppm)

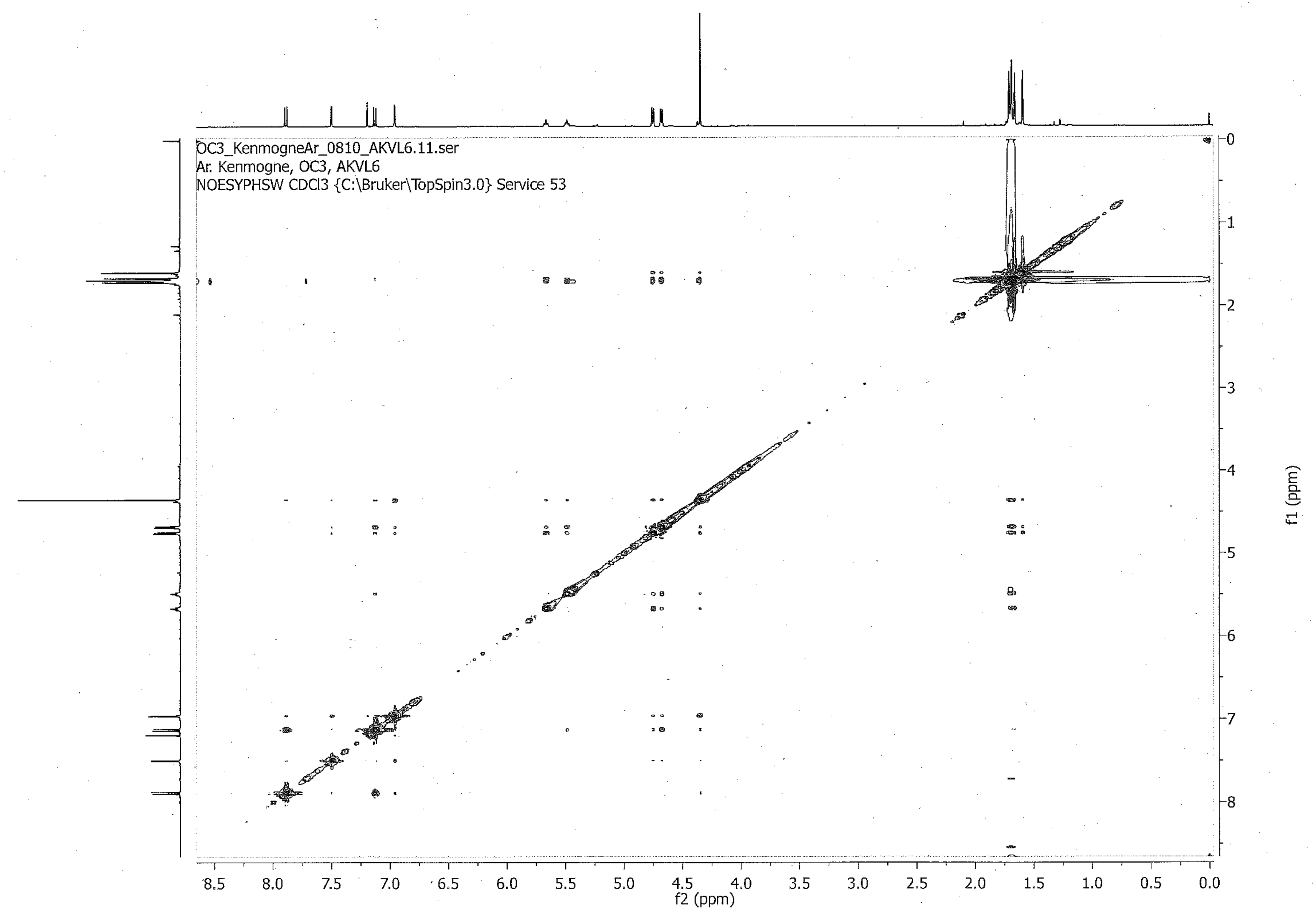

OC3_KenmogneAr_0810_AKVL6.11.ser
Ar. Kenmogne, OC3, AKVL6
NOESYPHSW CDCl3 {C:\Bruker\TopSpin3.0} Service 53
f2 (ppm)
f1 (ppm)

COMPOUND 2

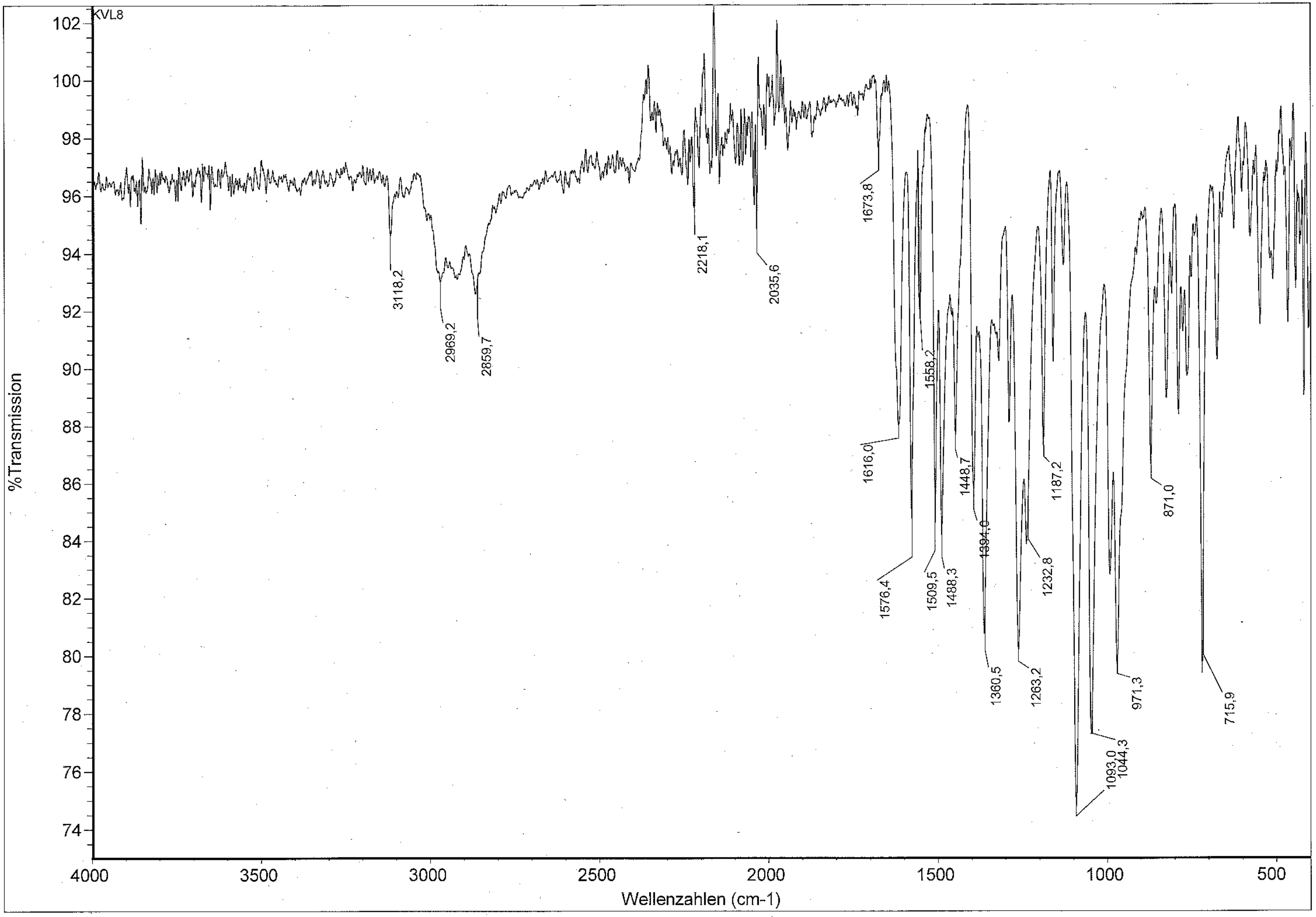
KVL8
%Transmission
Wellenzahlen (cm-1)
3118,2
2969,2
2859,7
2218,1
2035,6
1673,8
1616,0
1576,4
1558,2
1509,5
1488,3
1448,7
1394,0
1360,5
1263,2
1232,8
1187,2
1093,0
1044,3
971,3
871,0
715,9

File:MFB2016_109 Ident:48_52 SMO(1,7) PKD(7,3,7,0.50%,0.0,0.00%,F,F) SPEC(Heights,Centroid) Acq>
AutoSpec EI+ Voltage BpM:383 BpI:114236 TIC:359861 Flags:NORM
File Text:Ar. Kenmogne, OC3, AKVL8, HWP

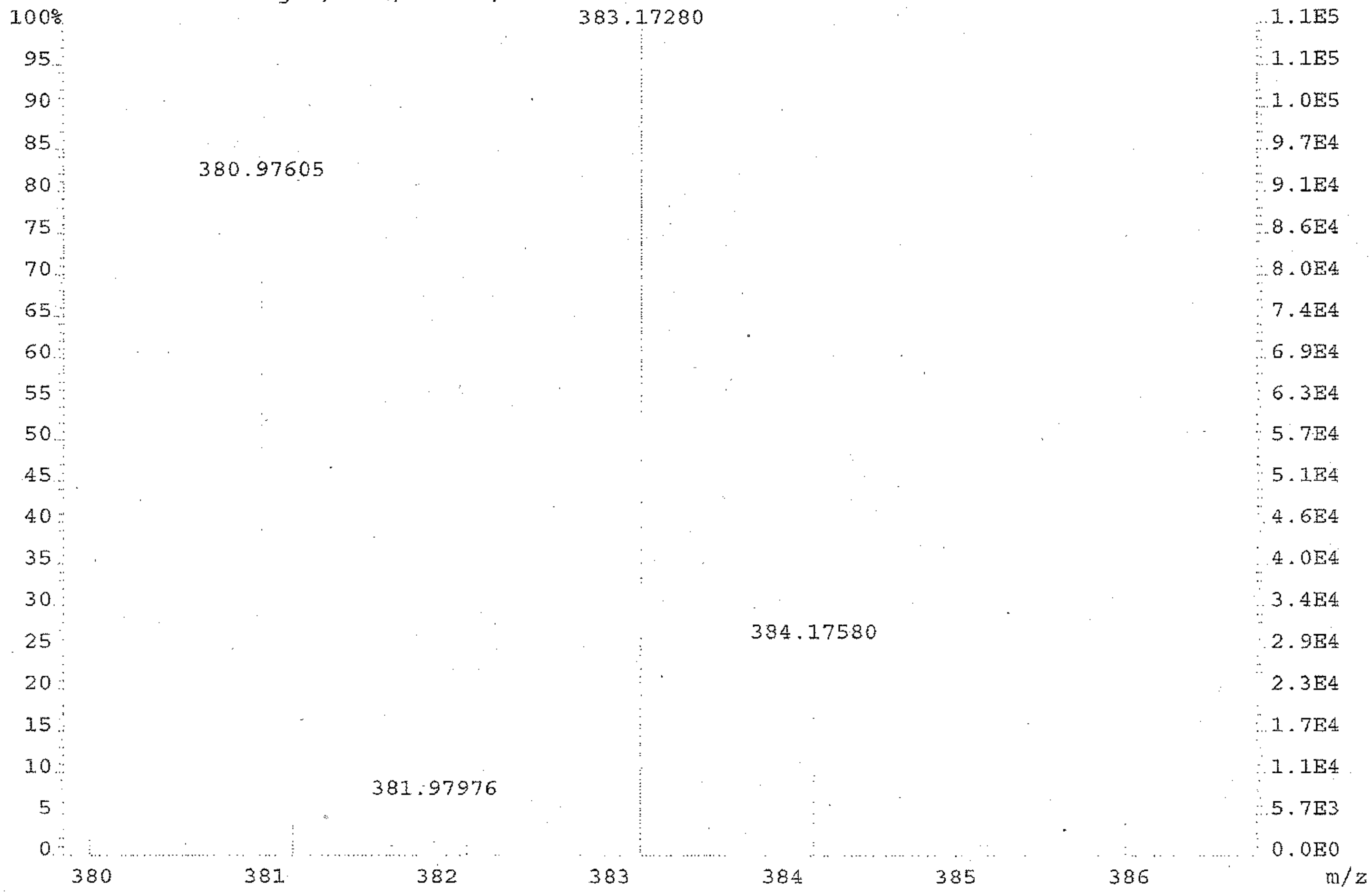

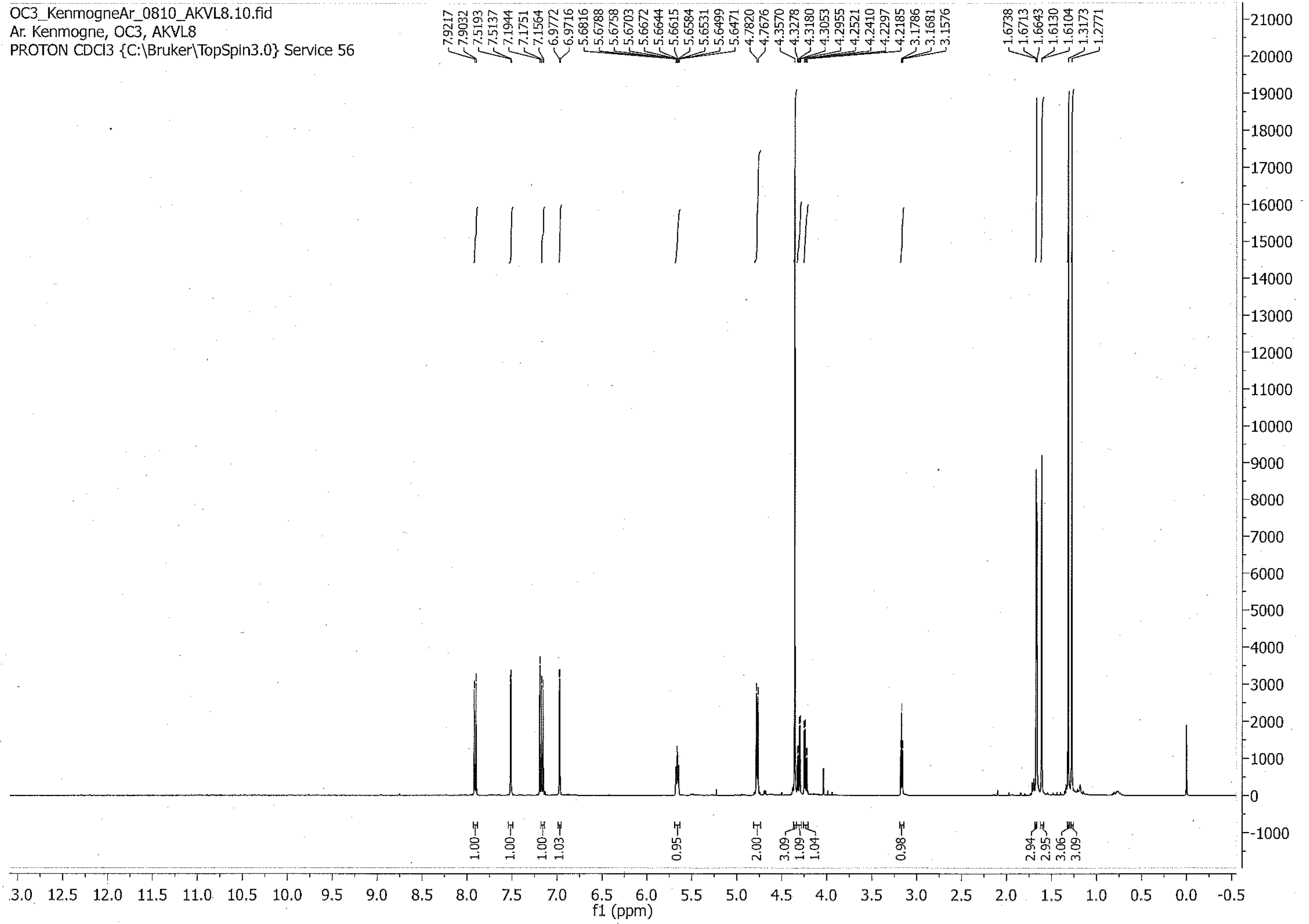
OC3_KenmogneAr_0810_AKVL8.10.fid
Ar. Kenmogne, OC3, AKVL8
PROTON CDCl3 {C:\Bruker\TopSpin3.0} Service 56
7.9217
7.9032
7.5193
7.5137
7.1944
7.1751
7.1564
6.9772
6.9716
5.6816
5.6788
5.6758
5.6703
5.6672
5.6644
5.6615
5.6584
5.6531
5.6499
5.6471
4.7820
4.7676
4.3570
4.3278
4.3180
4.3053
4.2955
4.2521
4.2410
4.2297
4.2185
3.1786
3.1681
3.1576
1.6738
1.6713
1.6643
1.6130
1.6104
1.3173
1.2771
1.00
1.00
1.00
1.03
0.95
2.00
3.09
1.09
1.04
0.98
2.94
2.95
3.06
3.09
f1 (ppm)

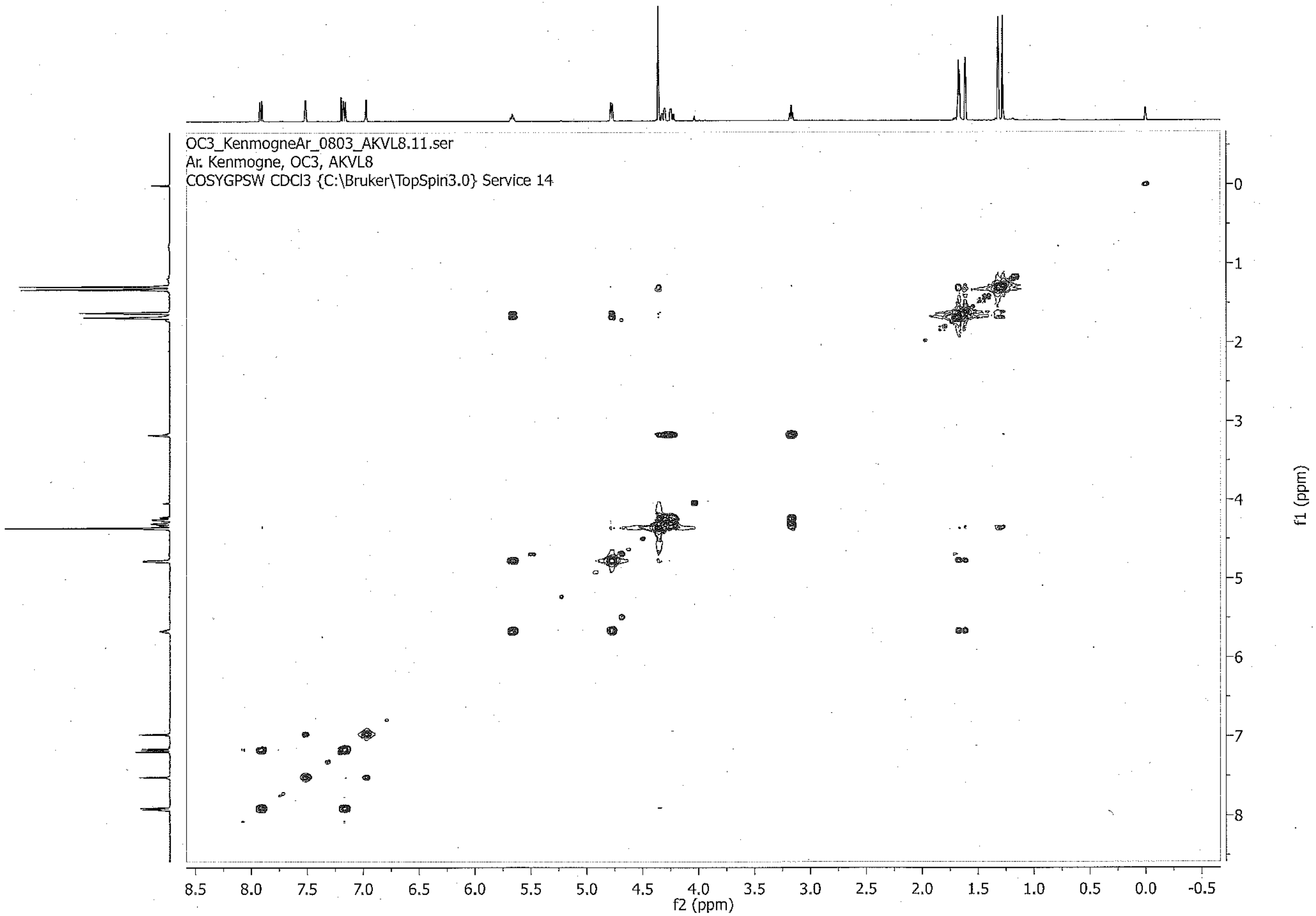

OC3_KenmogneAr_0803_AKVL8.11.ser
Ar. Kenmogne, OC3, AKVL8
COSYGPSW CDCl3 {C:\Bruker\TopSpin3.0} Service 14
f2 (ppm)
f1 (ppm)

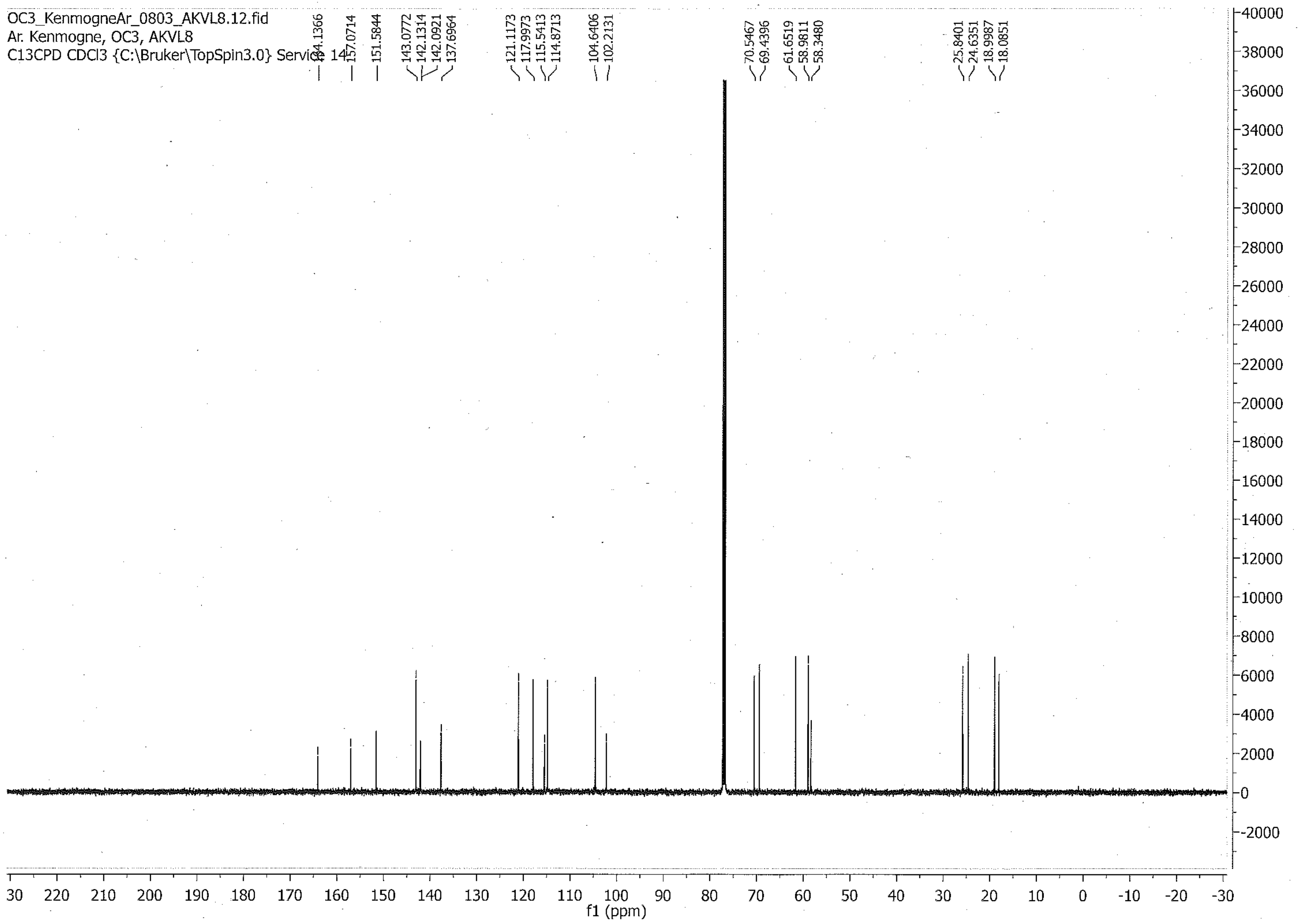
OC3_KenmogneAr_0803_AKVL8.12.fid
Ar. Kenmogne, OC3, AKVL8
C13CPD CDCl3 {C:\Bruker\TopSpin3.0} Service 14
4.1366
157.0714
151.5844
143.0772
142.1314
142.0921
137.6964
121.1173
117.9973
115.5413
114.8713
104.6406
102.2131
70.5467
69.4396
61.6519
58.9811
58.3480
25.8401
24.6351
18.9987
18.0851
40000
38000
36000
34000
32000
30000
28000
26000
24000
22000
20000
18000
16000
14000
12000
10000
8000
6000
4000
2000
0
-2000
30
220
210
200
190
180
170
160
150
140
130
120
110
100
90
80
70
60
50
40
30
20
10
0
-10
-20
-30
f1 (ppm)

OC3_KenmogneAr_0803_AKVL8.13.fid
Ar. Kenmogne, OC3, AKVL8
C13DEPT135 CDCl3 {C:\Bruker\TopSpin3.0} Service 14

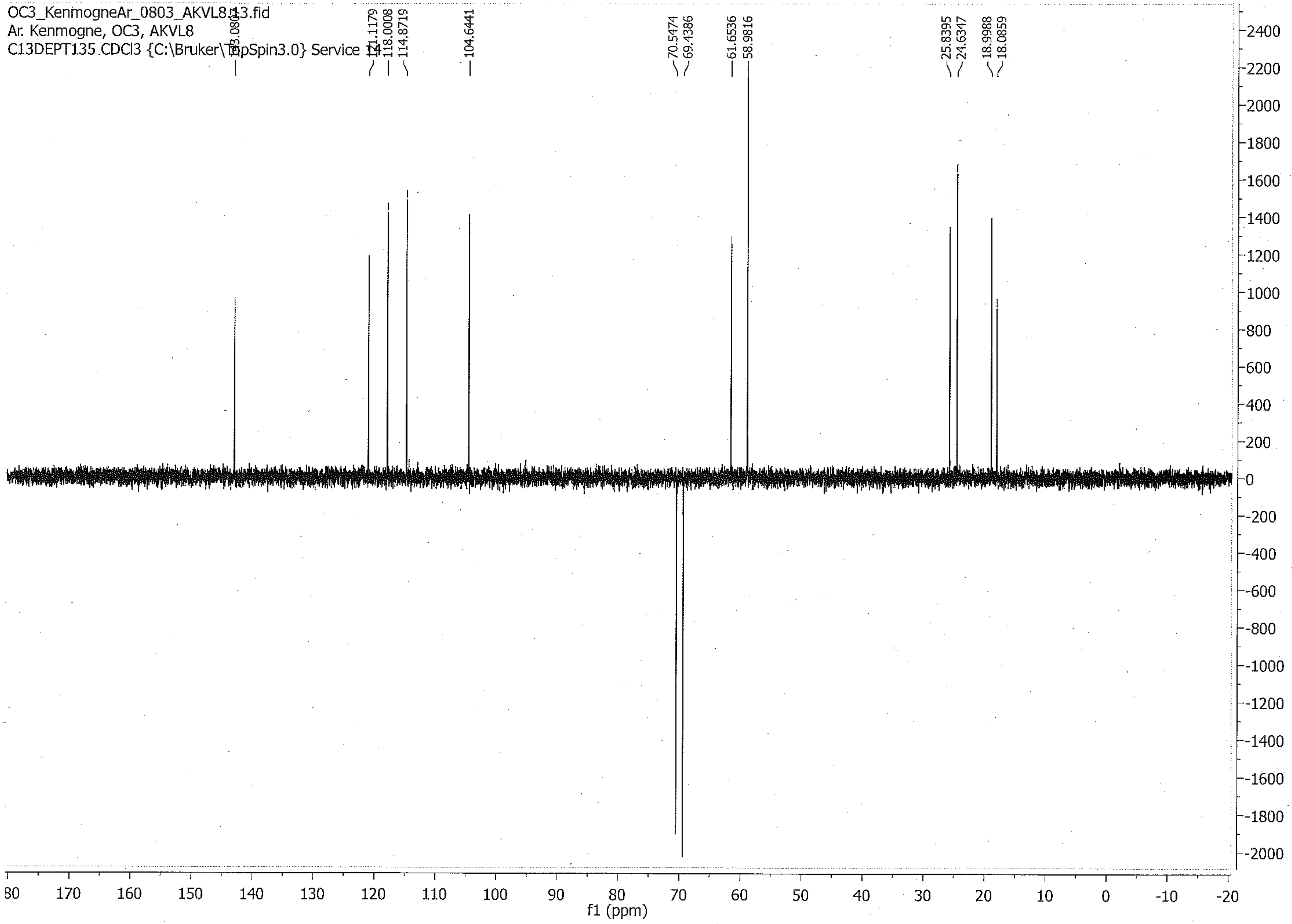

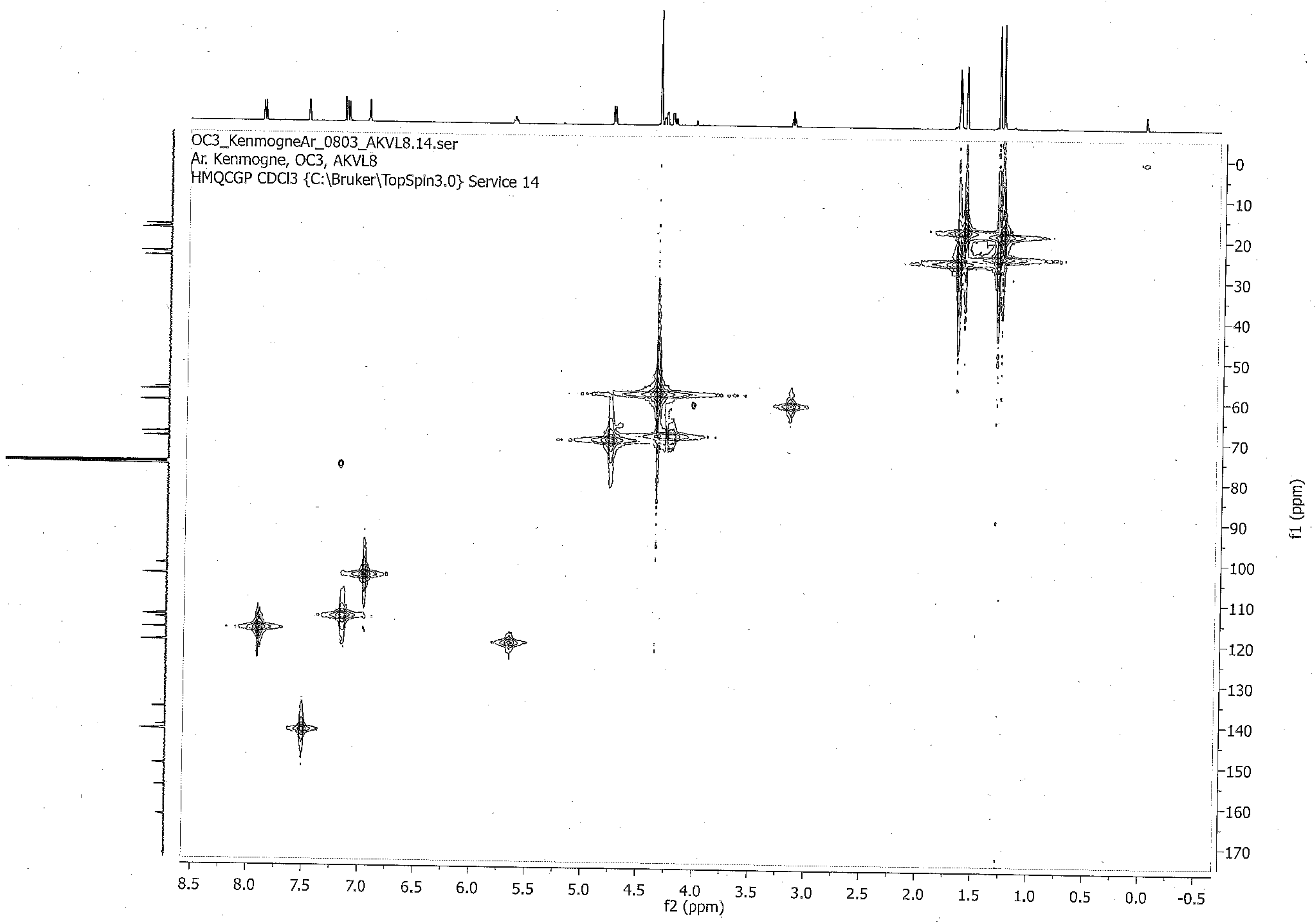

OC3_KenmogneAr_0803_AKVL8.14.ser
Ar. Kenmogne, OC3, AKVL8
HMQCGP CDCl3 {C:\Bruker\TopSpin3.0} Service 14
f2 (ppm)
f1 (ppm)

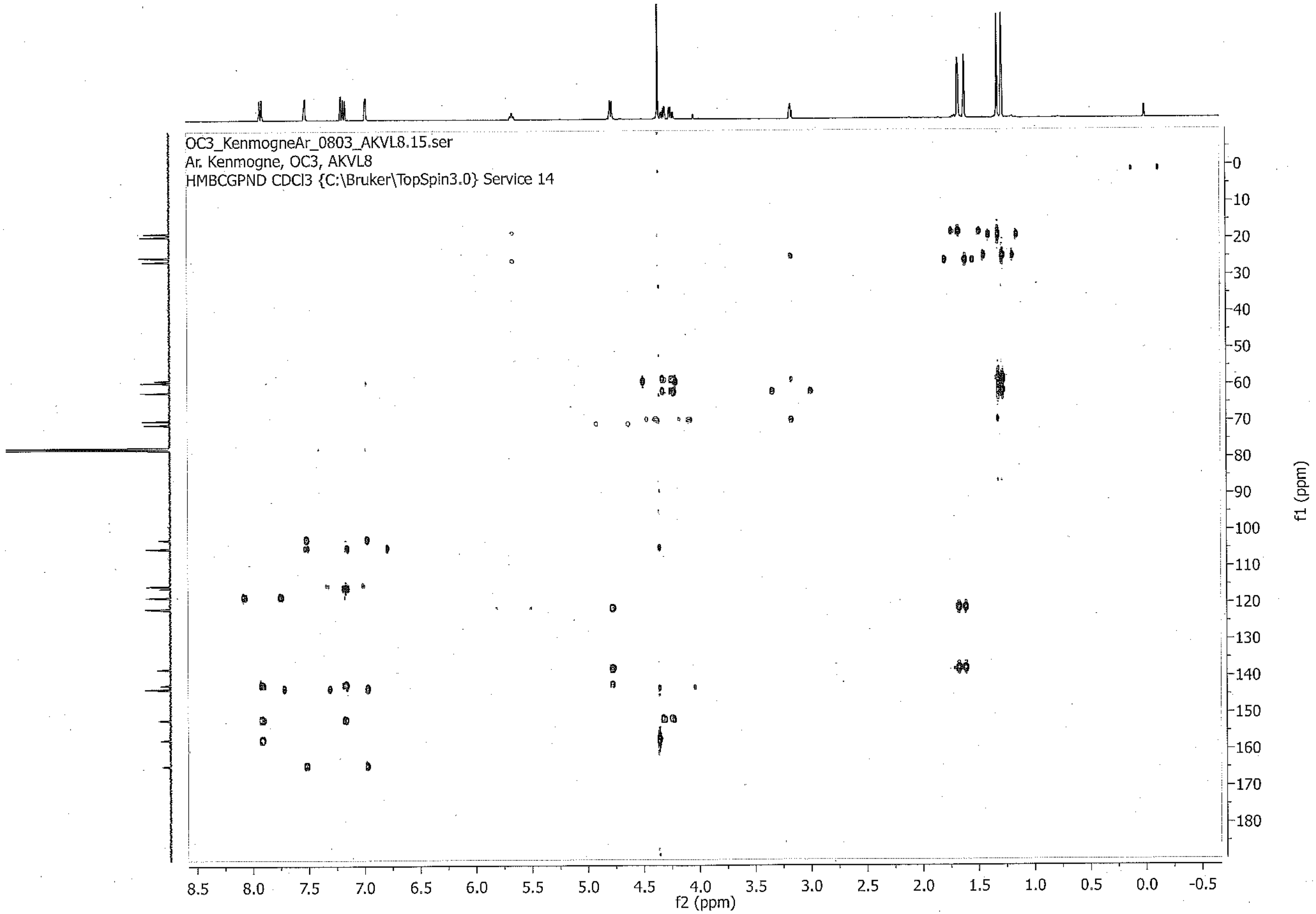
OC3_KenmogneAr_0803_AKVL8.15.ser
Ar. Kenmogne, OC3, AKVL8
HMBCGPND CDCl3 {C:\Bruker\TopSpin3.0} Service 14
f1 (ppm)
f2 (ppm)

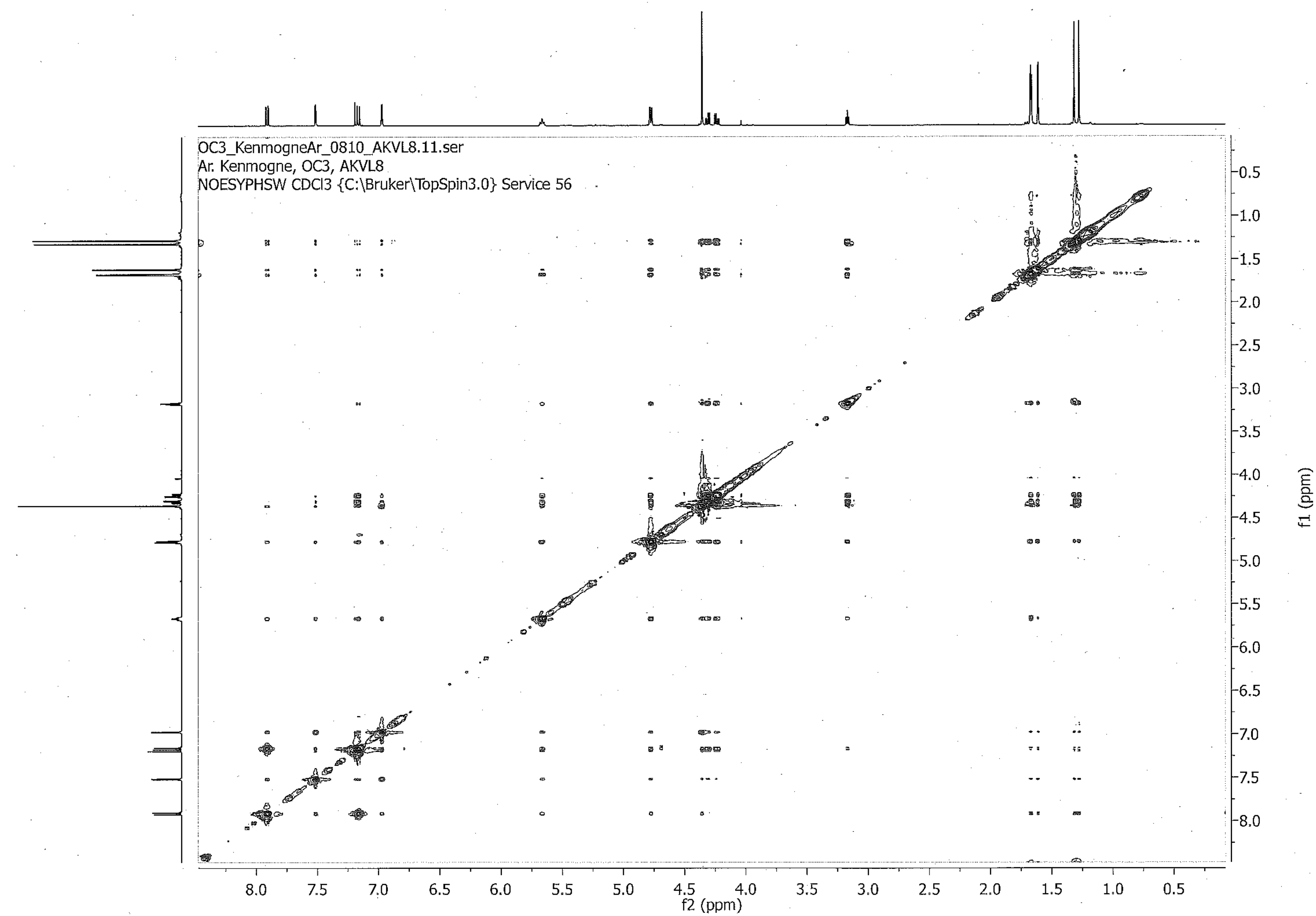

OC3_KenmogneAr_0810_AKVL8.11.ser
Ar. Kenmogne, OC3, AKVL8
NOESYPHSW CDCl3 {C:\Bruker\TopSpin3.0} Service 56
f2 (ppm)
f1 (ppm)

COMPOUND 3

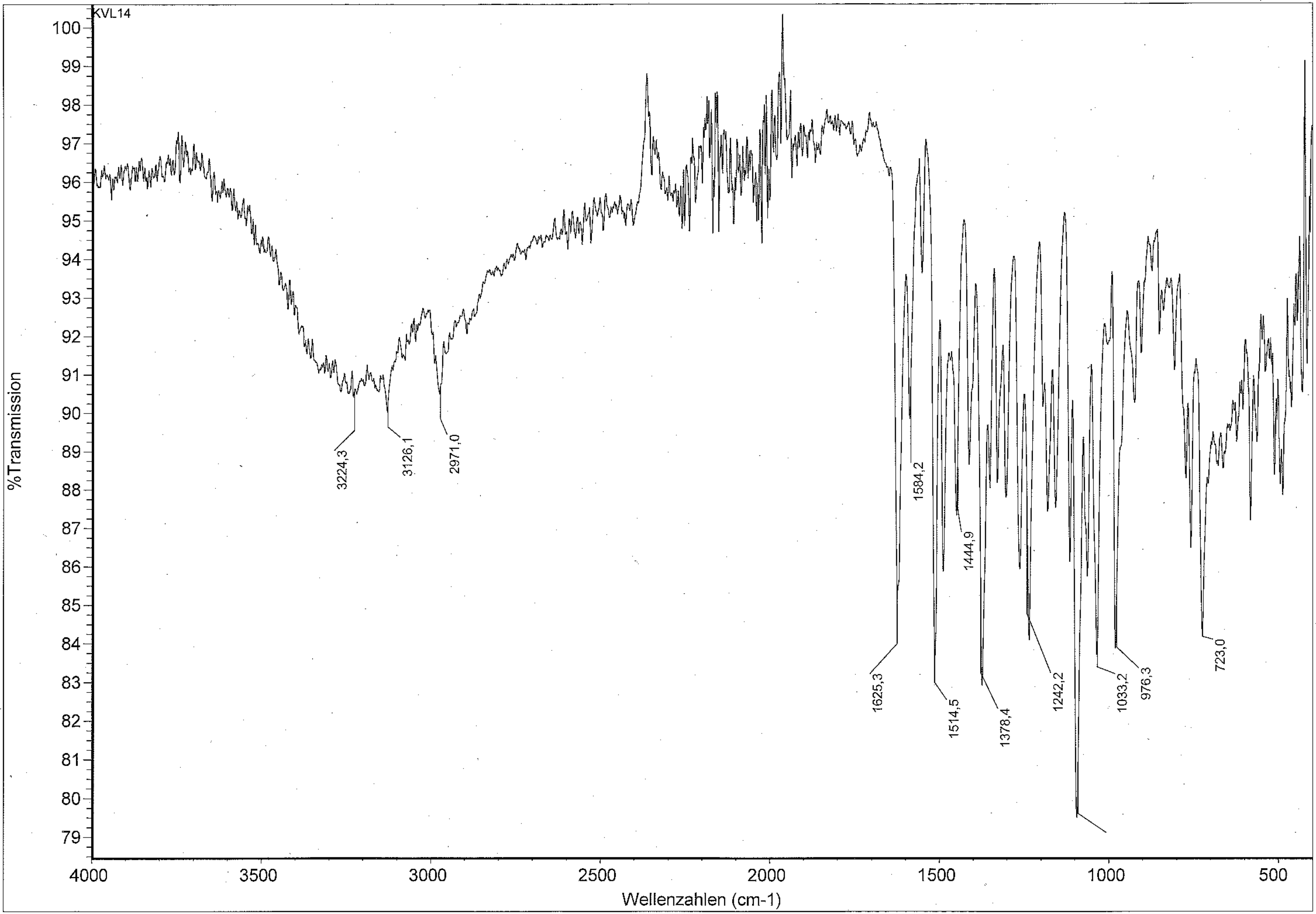
KVL14
%Transmission
100
99
98
97
96
95
94
93
92
91
90
89
88
87
86
85
84
83
82
81
80
79
3224,3
3126,1
2971,0
1584,2
1444,9
1625,3
1514,5
1378,4
1242,2
1033,2
976,3
723,0
4000
3500
3000
2500
2000
1500
1000
500
Wellenzahlen (cm-1)

File:MFB2016_123X Ident:40_50 SMO(1,7) PKD(7,3,7,0.50%,0.0,0.00%,F,F) SPEC(Heights,Centroid) Ac>
AutoSpec EI+ Voltage BpM:319 BpI:41690 TIC:159279 Flags:NORM
File Text:Ar. Kenmogne, OC3, AKVL14, HWP

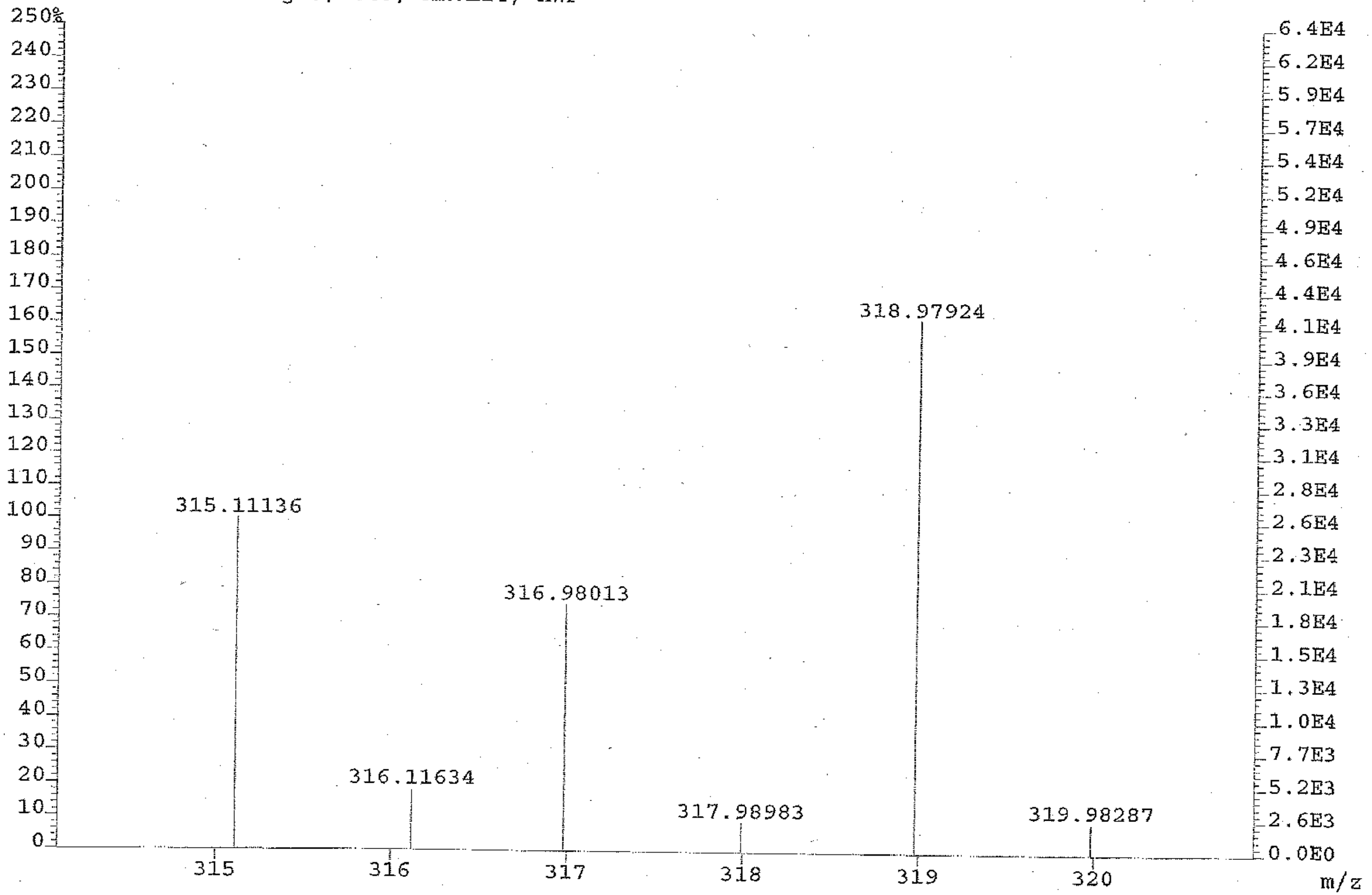

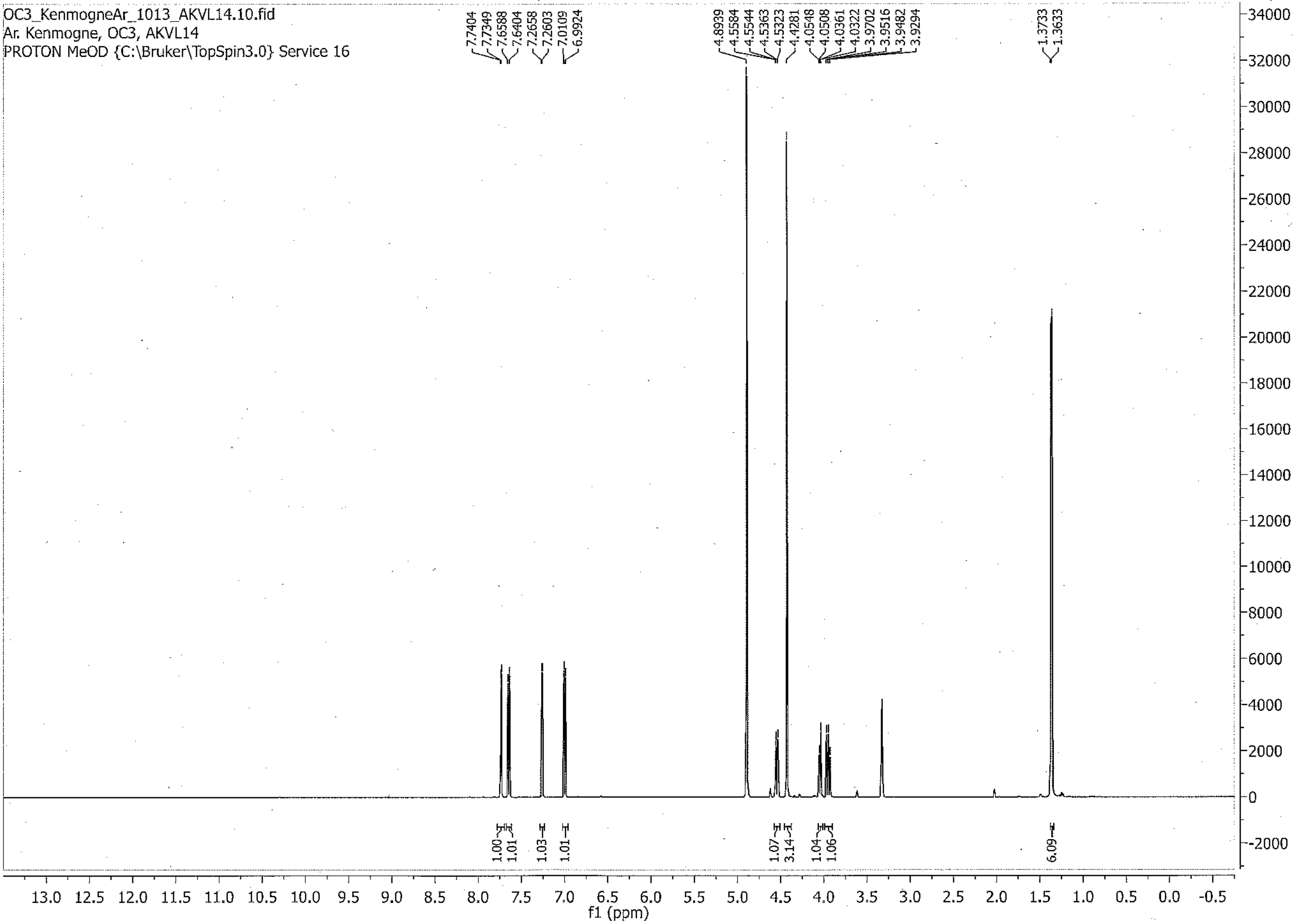

OC3_KenmogneAr_1013_AKVL14.10.fid
Ar. Kenmogne, OC3, AKVL14
PROTON MeOD {C:\Bruker\TopSpin3.0} Service 16
7.7404
7.7349
7.6588
7.6404
7.2658
7.2603
7.0109
6.9924
4.8939
4.5584
4.5544
4.5363
4.5323
4.4281
4.0548
4.0508
4.0361
4.0322
3.9702
3.9516
3.9482
3.9294
1.3733
1.3633
1.00
1.01
1.03
1.01
1.07
3.14
1.04
1.06
6.09
f1 (ppm)

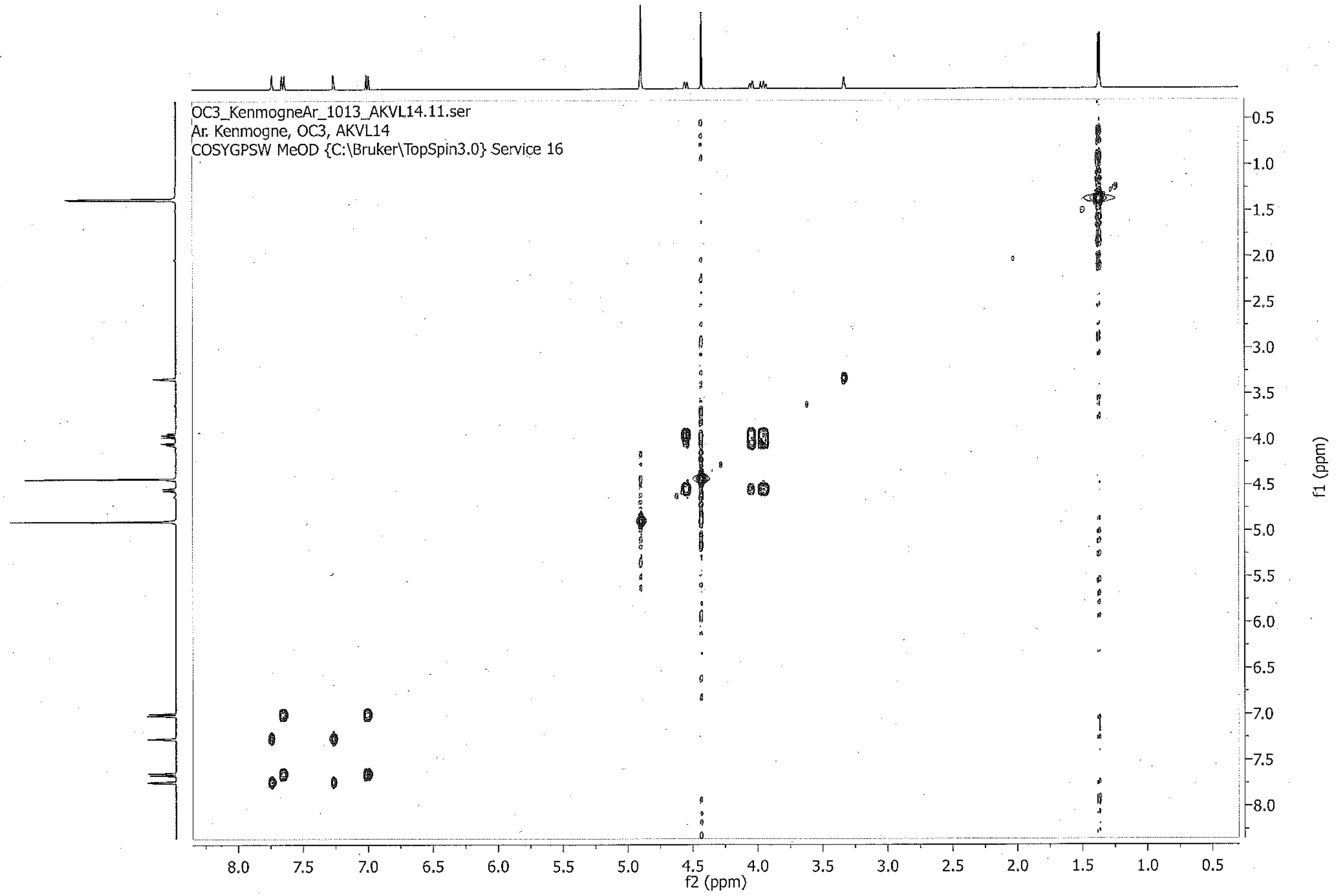
OC3_KenmogneAr_1013_AKVL14.11.ser
Ar. Kenmogne, OC3, AKVL14
COSYGPSW MeOD {C:\Bruker\TopSpin3.0} Service 16
8.0 7.5 7.0 6.5 6.0 5.5 5.0 4.5 4.0 3.5 3.0 2.5 2.0 1.5 1.0 0.5
f2 (ppm)
0.5 1.0 1.5 2.0 2.5 3.0 3.5 4.0 4.5 5.0 5.5 6.0 6.5 7.0 7.5 8.0
f1 (ppm)

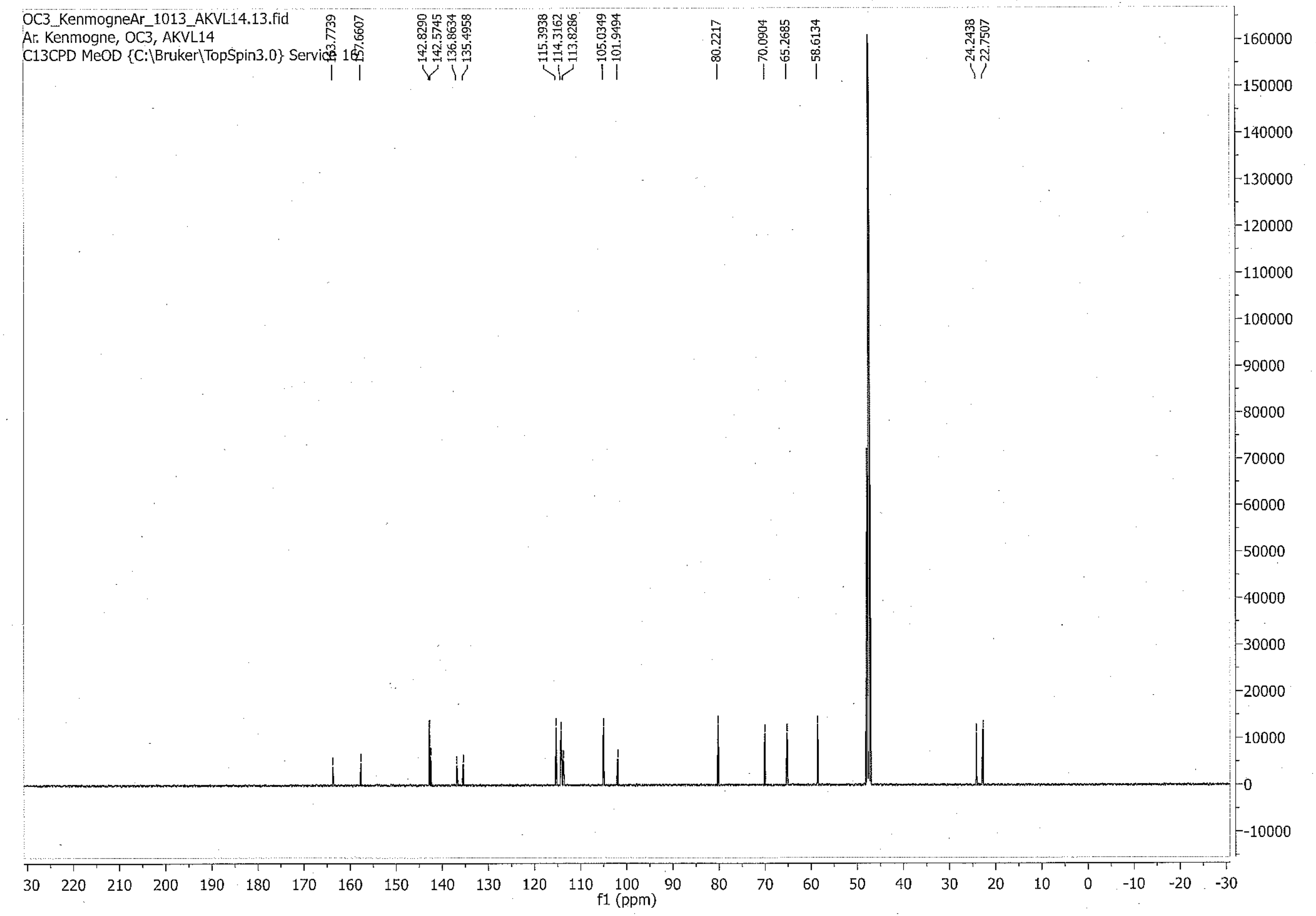

OC3_KenmogneAr_1013_AKVL14.13.fid
Ar. Kenmogne, OC3, AKVL14
C13CPD MeOD {C:\Bruker\TopSpin3.0} Service 16
163.7739
157.6607
142.8290
142.5745
136.8634
135.4958
115.3938
114.3162
113.8286
105.0349
101.9494
80.2217
70.0904
65.2685
58.6134
24.2438
22.7507
f1 (ppm)

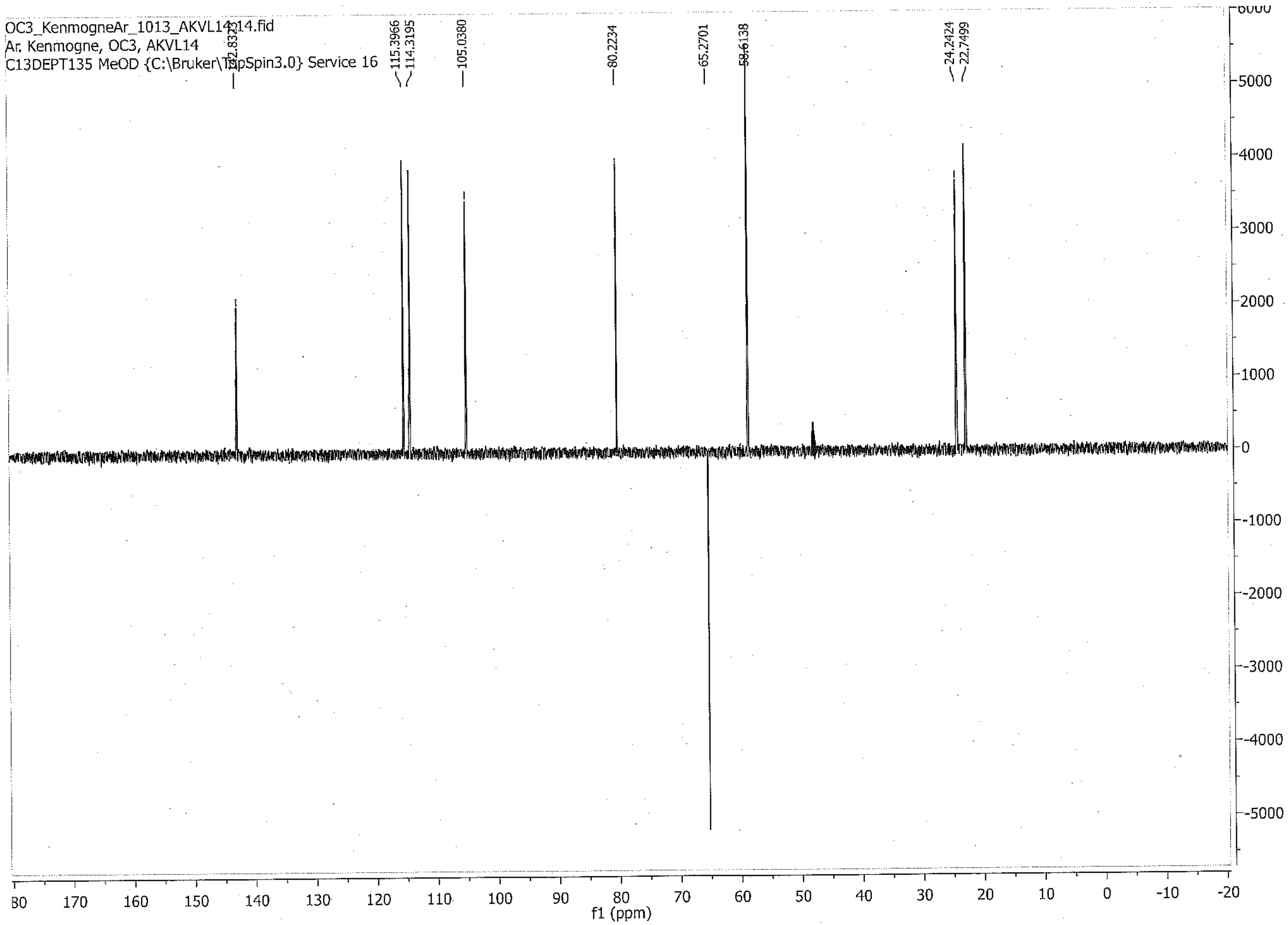

OC3_KenmogneAr_1013_AKVL14_14.fid
Ar. Kenmogne, OC3, AKVL14
C13DEPT135 MeOD {C:\Bruker\TopSpin3.0} Service 16
115.3966
114.3195
105.0380
80.2234
65.2701
58.6138
24.2424
22.7499
6000
5000
4000
3000
2000
1000
0
-1000
-2000
-3000
-4000
-5000
180 170 160 150 140 130 120 110 100 90 80 70 60 50 40 30 20 10 0 -10 -20
f1 (ppm)

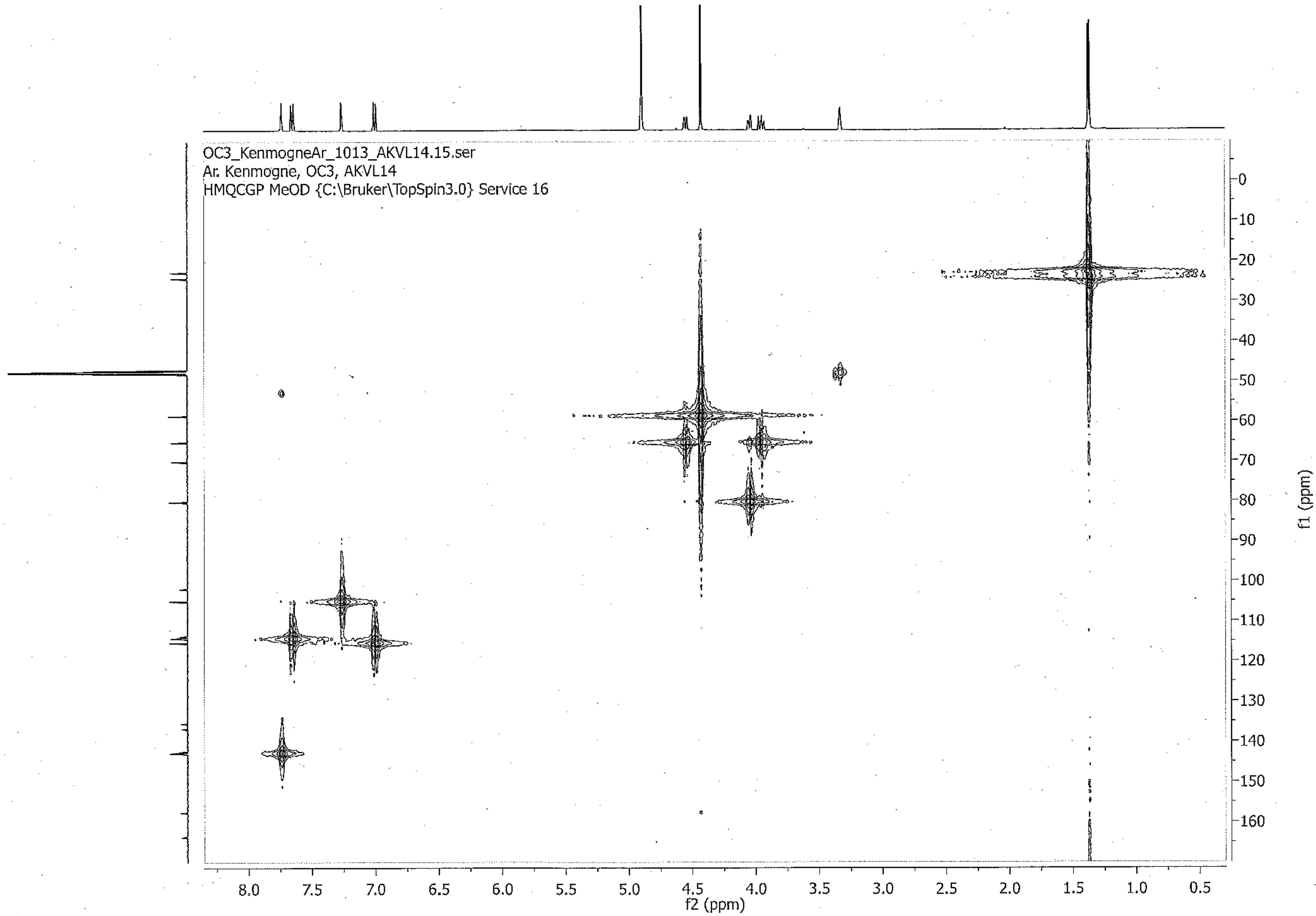
OC3_KenmogneAr_1013_AKVL14.15.ser
Ar. Kenmogne, OC3, AKVL14
HMQCGP MeOD {C:\Bruker\TopSpin3.0} Service 16
f2 (ppm)
f1 (ppm)
8.0 7.5 7.0 6.5 6.0 5.5 5.0 4.5 4.0 3.5 3.0 2.5 2.0 1.5 1.0 0.5
0 10 20 30 40 50 60 70 80 90 100 110 120 130 140 150 160

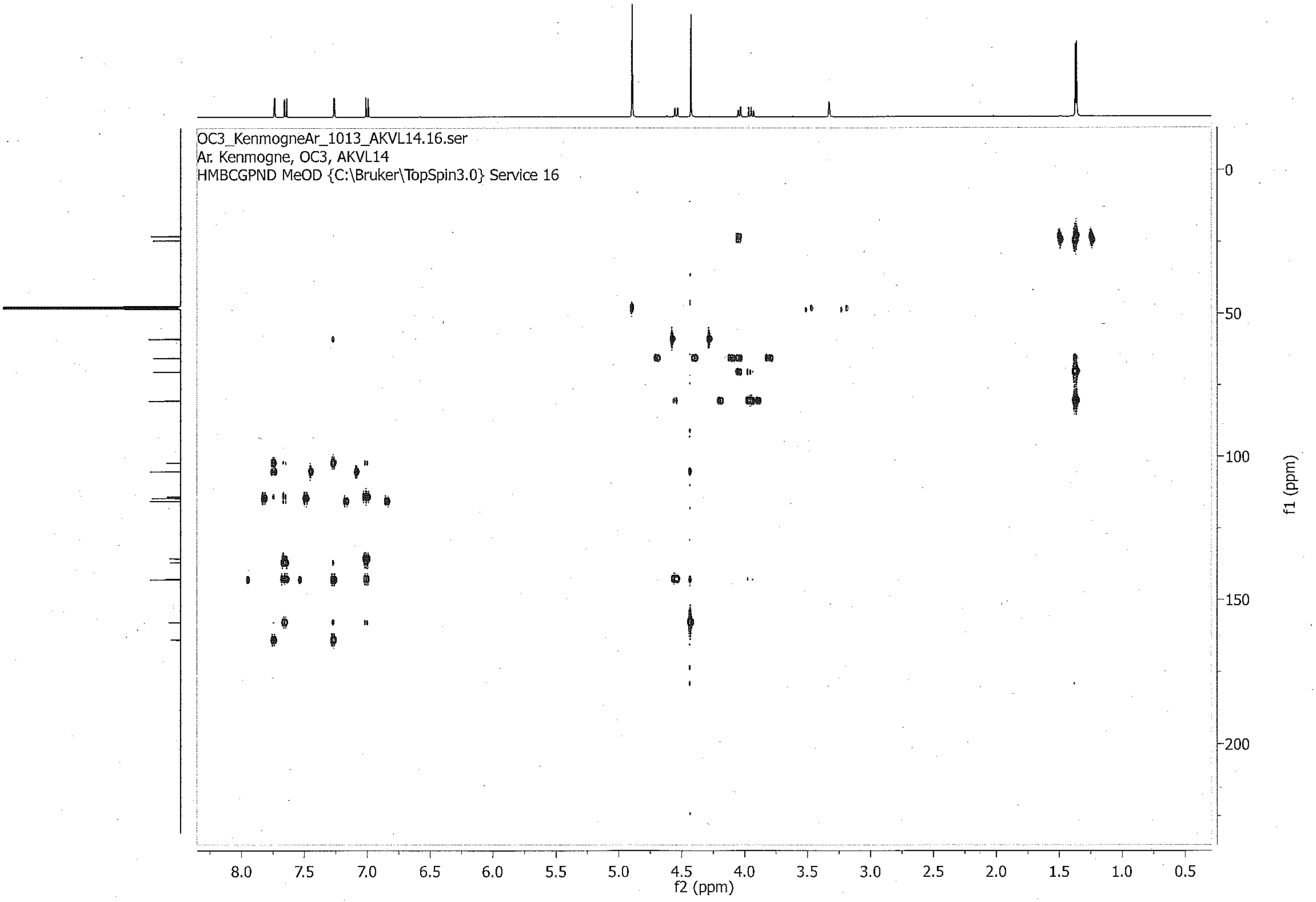
OC3_KenmogneAr_1013_AKVL14.16.ser
Ar. Kenmogne, OC3, AKVL14
HMBCGPND MeOD {C:\Bruker\TopSpin3.0} Service 16
f2 (ppm)
f1 (ppm)
8.0
7.5
7.0
6.5
6.0
5.5
5.0
4.5
4.0
3.5
3.0
2.5
2.0
1.5
1.0
0.5
0
50
100
150
200

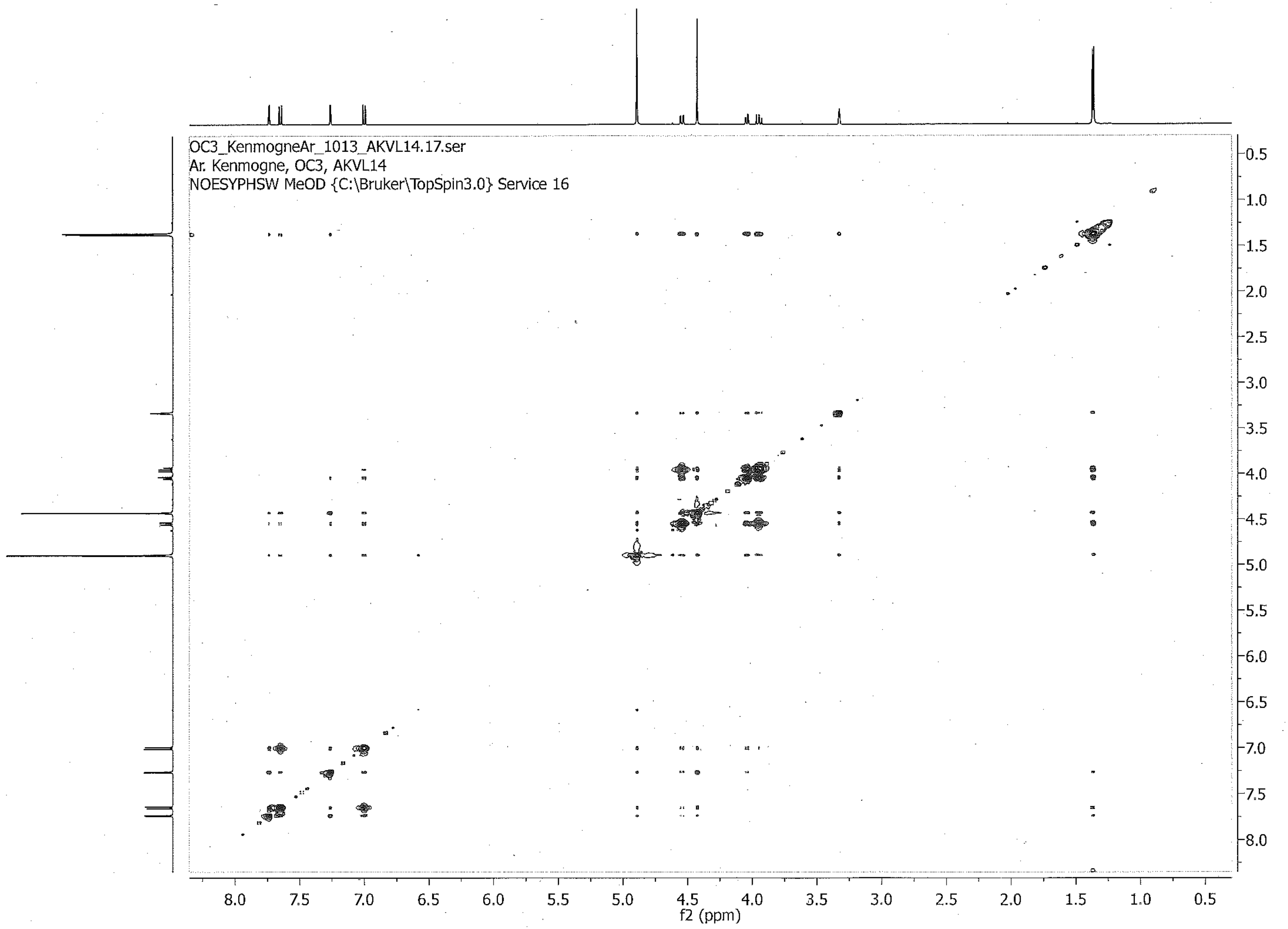
OC3_KenmogneAr_1013_AKVL14.17.ser
Ar. Kenmogne, OC3, AKVL14
NOESYPHSW MeOD {C:\Bruker\TopSpin3.0} Service 16
f2 (ppm)
f1 (ppm)
8.0
7.5
7.0
6.5
6.0
5.5
5.0
4.5
4.0
3.5
3.0
2.5
2.0
1.5
1.0
0.5
